# Biochemical Warfare on the Reef: The Role of Glutathione Transferases in Consumer Tolerance of Dietary Prostaglandins

**DOI:** 10.1371/journal.pone.0008537

**Published:** 2010-01-06

**Authors:** Kristen E. Whalen, Amy L. Lane, Julia Kubanek, Mark E. Hahn

**Affiliations:** 1 Biology Department, Woods Hole Oceanographic Institution, Woods Hole, Massachusetts, United States of America; 2 School of Chemistry and Biochemistry, Georgia Institute of Technology, Atlanta, Georgia, United States of America; 3 School of Biology, Georgia Institute of Technology, Atlanta, Georgia, United States of America; Purdue University, United States of America

## Abstract

**Background:**

Despite the profound variation among marine consumers in tolerance for allelochemically-rich foods, few studies have examined the biochemical adaptations underlying diet choice. Here we examine the role of glutathione *S*-transferases (GSTs) in the detoxification of dietary allelochemicals in the digestive gland of the predatory gastropod *Cyphoma gibbosum*, a generalist consumer of gorgonian corals. Controlled laboratory feeding experiments were used to investigate the influence of gorgonian diet on *Cyphoma* GST activity and isoform expression. Gorgonian extracts and semi-purified fractions were also screened to identify inhibitors and possible substrates of *Cyphoma* GSTs. In addition, we investigated the inhibitory properties of prostaglandins (PGs) structurally similar to antipredatory PGs found in high concentrations in the Caribbean gorgonian *Plexaura homomalla*.

**Principal Findings:**

*Cyphoma* GST subunit composition was invariant and activity was constitutively high regardless of gorgonian diet. Bioassay-guided fractionation of gorgonian extracts revealed that moderately hydrophobic fractions from all eight gorgonian species examined contained putative GST substrates/inhibitors. LC-MS and NMR spectral analysis of the most inhibitory fraction from *P. homomalla* subsequently identified prostaglandin A_2_ (PGA_2_) as the dominant component. A similar screening of commercially available prostaglandins in series A, E, and F revealed that those prostaglandins most abundant in gorgonian tissues (e.g., PGA_2_) were also the most potent inhibitors. *In vivo* estimates of PGA_2_ concentration in digestive gland tissues calculated from snail grazing rates revealed that *Cyphoma* GSTs would be saturated with respect to PGA_2_ and operating at or near physiological capacity.

**Significance:**

The high, constitutive activity of *Cyphoma* GSTs is likely necessitated by the ubiquitous presence of GST substrates and/or inhibitors in this consumer's gorgonian diet. This generalist's GSTs may operate as ‘all-purpose’ detoxification enzymes, capable of conjugating or sequestering a broad range of lipophilic gorgonian compounds, thereby allowing this predator to exploit a range of chemically-defended prey, resulting in a competitive dietary advantage for this species.

## Introduction

Glutathione *S*-transferases (GSTs, EC 2.5.1.18) comprise a large superfamily of enzymes whose soluble members primarily function as detoxification enzymes, facilitating the conjugation of a diverse array of hydrophobic electrophilic xenobiotics by the nucleophilic attack of glutathione [1]. GSTs have long been known as important components of cellular defense mechanisms in mammalian systems [2] and recent studies are revealing their significance in mediating allelochemical tolerance in invertebrate-host interactions (reviewed in [3]).

The diversity of GST isoforms and their capacity to detoxify allelochemicals in consumers has been correlated with diet breadth [4]–[6]. For example, in a survey of GST isoforms from five lepidopteran species, specialist herbivores expressed only one major GST isoform, while generalists expressed multiple forms [7]. Furthermore, GST isoforms purified from polyphagous herbivores that regularly consume isothiocyanate-containing cruciferous plants were able to metabolize a broader range of isothiocyanate allelochemicals in comparison to GSTs from specialists that did not consume crucifers and lacked the ability to conjugate isothiocyanates [4]. These findings imply that the evolution of generalist GST forms favors promiscuous catalytic activity presumably needed to cope with the breadth of dietary toxins encountered; this mirrors results for other consumer counter-defense proteins (i.e., cytochrome P450s) whereby more catalytically flexible detoxification enzymes may promote a greater degree of polyphagy [8]. Moreover, while polyphagous insects have little to no ability to increase their GST expression upon allelochemical exposure, they often possess a higher constitutive level of GST activity in comparison to oligophagous and monophagous species [4], [9]. The difference in constitutive and inducible GST expression in consumers may ultimately be a reflection of the non-specific role of GSTs as antioxidant enzymes. GSTs are known to be under regulatory control by antioxidant response elements found in their promoters [2]. Due to the diverse range of dietary pro-oxidants encountered in polyphagous species [9], sustained transcriptional activation of GST enzymes by dietary compounds may result in the near maximal expression of GST enzymes [4]. Consequently, possessing constitutively expressed GST enzymes that are catalytically versatile may confer a selective advantage to those consumers that regularly encounter unpredictable host chemistry.

Similar to their terrestrial counterparts, marine consumers that regularly feed on allelochemically-rich prey may have evolved a parallel suite of biochemical resistance mechanisms (reviewed in [10], [11]). The induction or high constitutive activity of GSTs seen in several marine molluscs after allelochemical exposure has been suggested as a protective mechanism against dietary intoxication [12]–[18]. High cytosolic GST activity was observed from the digestive gland of the generalist marine gastropod *Cyphoma gibbosum* [14], [19], which feeds solely on a diet of chemically-defended gorgonians [20], and whose GST activity levels rival those of terrestrial invertebrates that feed solely on allelochemically-rich prey [21], [22]. This gastropod predator utilizes three families of gorgonian corals as hosts and in doing so encounters a range of lipophilic allelochemicals that includes diterpenes, sesquiterpenes, acetogens, highly-functionalized steroids and eicosanoids [23]–[25].

One gorgonian diet, *Plexaura homomalla*, has been suggested to be a favorite of *C. gibbosum* [26], [27] even though this gorgonian is known to contain impressive quantities of the cyclopentenone prostaglandin, PGA_2_ [28]–[32], which is known to serve as a feeding deterrent against generalist reef predators [33]–[35]. Perhaps related to their anti-predatory properties in marine systems, the cytotoxic nature of cyclopentenone prostaglandins occurs, in part, due to the reactive α,β-unsaturated carbonyl group in the cyclopentenone ring, which can undergo nucleophilic addition with electrophilic moieties, resulting in protein and DNA adduct formation [36]–[39]. Cyclopentenone prostaglandins of the A and J series have been shown to be inducers of GST enzymatic activity as well as mRNA expression in mammalian [40], [41] and invertebrate cell lines [42]. Furthermore, certain vertebrate alpha-, mu-, and pi-class GSTs were found to enhance PGA_2_ conjugation with glutathione, suggesting that the overexpression of GST forms could modulate the cytotoxic effects of cyclopentenone prostaglandins [37]. Mammalian GSTs also have the ability to non-catalytically bind lipophilic, amphipathic ligands, including PGJ_2_, via noncovalent interactions, which effectively sequester these ligands in the cytosol away from their nuclear targets (i.e., peroxisomal proliferator-activated receptor, PPAR) [43]. Given that *C. gibbosum* neither avoids *P. homomalla* nor adjusts its feeding rates to reduce toxin exposure [26], this snail likely possesses effective detoxification mechanisms, possibly GST-mediated, to contend with the high concentrations of dietary prostaglandins.

In a previous study, proteomic analysis of affinity-purified cytosolic GST fractions from *C. gibbosum* revealed that two major GST mu-class isoforms were responsible for the high GST activity observed in the digestive gland [19]. Here, in a controlled laboratory feeding study, we allowed snails to feed ad libitum for four days on one of seven gorgonian species or a diet devoid of gorgonians, and monitored GST activity levels and isoform expression in response to the different suites of gorgonian allelochemicals. To investigate whether gorgonian extracts contained possible substrates for *Cyphoma* GSTs, we used a bioassay-guided fractionation approach, screening gorgonian crude organic extracts and subsequent fractions of different polarities for their ability to inhibit the 1-chloro-2,4-dinitrobenzene (CDNB)-conjugating activity of GST. Selected HPLC fractions found to inhibit GST activity were further characterized by ^1^NMR and LC-MS spectral analyses to identify possible bioactive compounds. In addition, a series of commercially-available prostaglandins representing a range of eicosanoids previously described from *P. homomalla* were examined for their ability to inhibit *Cyphoma* GST activity.

## Results

### Gorgonian Dietary Influence on GST Activity and Subunit Expression

**Figure 1:**
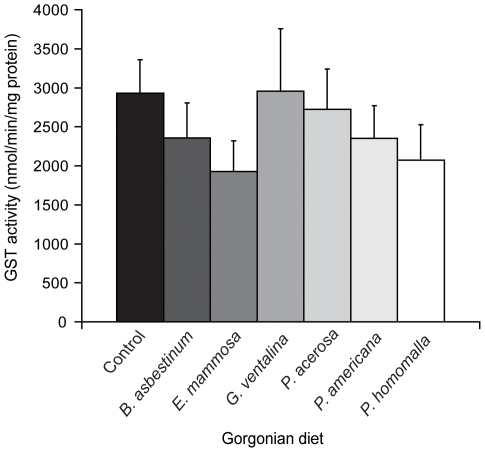
Digestive gland GST activity does not differ with snail diet. Bars represent the mean GST activity (±SE) of snails feeding on the control diet (n = 12 snails) or one of six gorgonian diets – *B. asbestinum* (n = 4), *E. mammosa* (n = 4), *G. ventalina* (n = 4), *P. acerosa* (n = 4), *P. americana* (n = 7) and *P. homomalla* (n = 4). The reaction mixture contained 2 µg of cytosolic protein in 0.1 M KPO_4_ buffer, pH 7.5, containing 1 mM GSH and 1 mM CDNB at 25°C. GST activity did not differ among snail diets (ANOVA, *P* = 0.687) or among snails feeding on the control diet collected from different reefs (ANOVA, *P* = 0.109).

GST specific activity levels measured from digestive gland cytosolic preparations ranged from 1930 to 2957 nmol min^−1^mg protein^−1^. GST activity levels were within the range reported by Vrolijk and Targett [14], but did not differ significantly between snail diets (Figure 1). HPLC separation of affinity-purified GSTs identified fourteen unique peaks (see Figure S1). HPLC peak 1 was previously identified as a theta-class GST, while HPLC peaks 2 thru 14 were identified as mu-class GST subunits [44]. HPLC peaks 4 and 8 represented the majority of expressed GST subunits at 25% and 68%, respectively. The relative proportion of each GST subunit, represented by separate HPLC peaks and calculated based on HPLC peak area, did not differ significantly as a function of gorgonian diet when expressed either as percent of all subunits present or when normalized to the amount of affinity-purified GST sample injected on to the HPLC column (see Figure S2). These results indicate that while GST activity is constitutively expressed at high levels in *Cyphoma* digestive gland, both GST activity and subunit abundance are unaffected by gorgonian diet.

### Inhibition of GST Activity by Gorgonian Extracts

**Figure 2:**
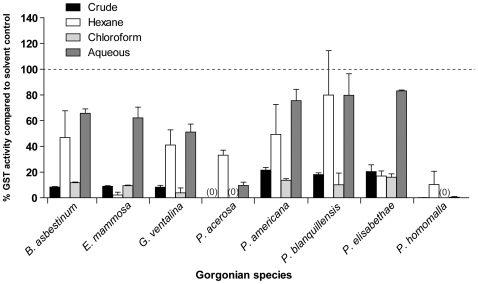
Gorgonian crude and semi-purified extracts inhibit *Cyphoma* GST activity. Bars represent the mean (±SE) percent GST activity remaining after addition of gorgonian compounds compared to solvent controls. Reaction mixture consisted of 2 µg cytosolic protein in 0.1 M potassium phosphate buffer, pH 7.5, containing 1 mM GSH, 1 mM CDNB at 25°C with <0.01% (v/v) solvent. Reactions were performed in duplicate with digestive gland crude cytosolic preparations from two snails. Crude extracts from eight gorgonian species were tested at 5% natural vol. concentration (NC) for their ability to inhibit crude cytosolic GST activity. Hexane and aqueous soluble extracts were tested at 25% NC. Chloroform soluble fractions were tested at 25% NC with the following exceptions: *G. ventalina*, *P. acerosa*, and *P. homomalla* were tested at 12% NC. A zero indicates complete inhibition of GST activity by the gorgonian extracts and the dotted line indicates no difference from solvent controls.

Crude organic extracts from *B. asbestinum*, *E. mammosa*, *G. ventalina*, *P. acerosa*, *P. americana*, *P. blanquillensis*, *P. elisabethae*, and *P. homomalla* tested at 5% natural volumetric concentration (NC) inhibited ≥70% of the GST activity in *Cyphoma* digestive gland cytosol compared to solvent controls (Figure 2). Chloroform-soluble fractions from all gorgonian species examined consistently showed ≥80% inhibition of GST activity compared to controls. Aqueous fractions generally displayed minor inhibitory effects, with the exception of fractions from *P. acerosa* and *P. homomalla*, which inhibited GST activity by 85% and 99%, respectively. Hexane-soluble fractions exhibited intermediate and wide-ranging inhibitory effects depending on gorgonian species (Figure 2).

To further investigate the source of the putative gorgonian GST substrates/inhibitors, we used HPLC to fractionate the chloroform-soluble extracts of each gorgonian and tested their ability to inhibit the activity of affinity-purified *Cyphoma* GSTs. For all eight gorgonian species, HPLC fraction 1 (compounds eluting from 3 to 6 mins) exhibited the greatest inhibitory activity, causing >80% GST inhibition at 10% NC compared to paired solvent controls (see Figure S3). Inspection of HPLC chromatograms for all gorgonian species indicated that HPLC fraction 1 consisted of a mixture of compounds. For four of these gorgonian species (*B. asbestinum*, *E. mammosa*, *P. acerosa*, and *P. homomalla*) affinity-purified GST activity was completely inhibited by HPLC fraction 1 at 10% NC. Diluting HPLC fraction 1 from *B. asbestinum*, *E. mammosa*, *P. acerosa*, and *P. homomalla* to 0.05% NC decreased the inhibitory effect of the compound(s); however, in all cases diluted fractions still retained the ability to inhibit >65% of affinity-purified GST activity compared to solvent controls.

### Identification of Prostaglandins in *P. homomalla* Extracts

Because *P. homomalla* is a favored diet of *Cyphoma* [26], [27], [45] and possesses extracts determined to significantly inhibit the CDNB-conjugating activity of *Cyphoma* GSTs, we focused our subsequent efforts on elucidating the compound(s) responsible for this inhibition. LC-MS and ^1^H-NMR spectral analysis of HPLC fraction 1 from the chloroform-soluble extract revealed the presence of PGA_2_. The ^1^H-NMR spectrum showed a 3H triplet at 0.85 ppm, consistent with the presence of a terminal methyl group in prostaglandins (see Figure S4). The mass spectrum of HPLC fraction 1 displayed a parent ion at *m/z* 333 with fragment peaks at *m/z* 315, 271, 233, and 189, characteristic of PGA_2_ (see Figure S5).

For HPLC fraction 2 from *P. homomalla*, the LC-MS signal at ∼2.5 min showed an *m/z* 371.2, which corresponds to [M+Na]^+^ of 5Z and 5*E*-prostaglandin B_2_ methyl ester [46] (see Figure S6). The ^1^H NMR spectrum of *P. homomalla* HPLC fraction 2 showed a dominant prostaglandin-like compound that matched the literature values (ca. <0.25 ppm difference between literature and experimental values) for 5*Z* and 5*E*-prostaglandin B_2_ methyl ester; however, this compound showed a doublet of doublets at 7.6 ppm which was substantially further downfield than expected. The dominant compound in HPLC fraction 2 also displayed a singlet at 2.0 ppm, suggestive of acylation. A second peak at ∼16.6 min in the LC-MS signal for HPLC fraction 2 showed an *m*/*z* 413.2, which corresponds to [M+Na]^+^ of either 5*Z* or 5*E*-acetyl-prostaglandin B_2_ methyl ester [46]. ^1^H NMR spectral data for the dominant compound were in agreement with those from 5*Z* and 5*E*-acetyl-prostaglandin B_2_ methyl ester, with the major exception of the signal at 7.6 ppm, which is further downfield than expected for these known compounds. The *m/z* of 413.2 also corresponds to 15-epi-prostaglandin A_2_ diester, whose ^1^H NMR spectrum matched very closely to the dominant compound in HPLC fraction 2, including the signal at 7.6 ppm. However, further comparison was made difficult due to an absence of a complete set of NMR spectral data in the literature for 15-epi-prostaglandin A_2_ diester [30]. Overall, ^1^H NMR and LC-MS spectral data support the presence of a dominant prostaglandin-derivative in *P. homomalla* HPLC fraction 2; however, because this fraction is a mixture of compounds, the exact identity cannot be established.

Quantification of PGA_2_ from *P. homomalla* HPLC fraction 1 by LC-MS revealed an approximate whole tissue concentration of 1.6 mM (or 530 µg PGA_2_/mL of wet gorgonian tissue). HPLC fraction 2 (eluting at 6–9 mins) from *P. homomalla* showed a selected ion recording (*m/z* 333.3) at the expected retention time; however, peak intensities were below the limit of quantification and subsequent NMR spectral analysis indicated that PGA_2_ was not present.

### Inhibition of GST Activity by Pure Prostaglandins

**Figure 3:**
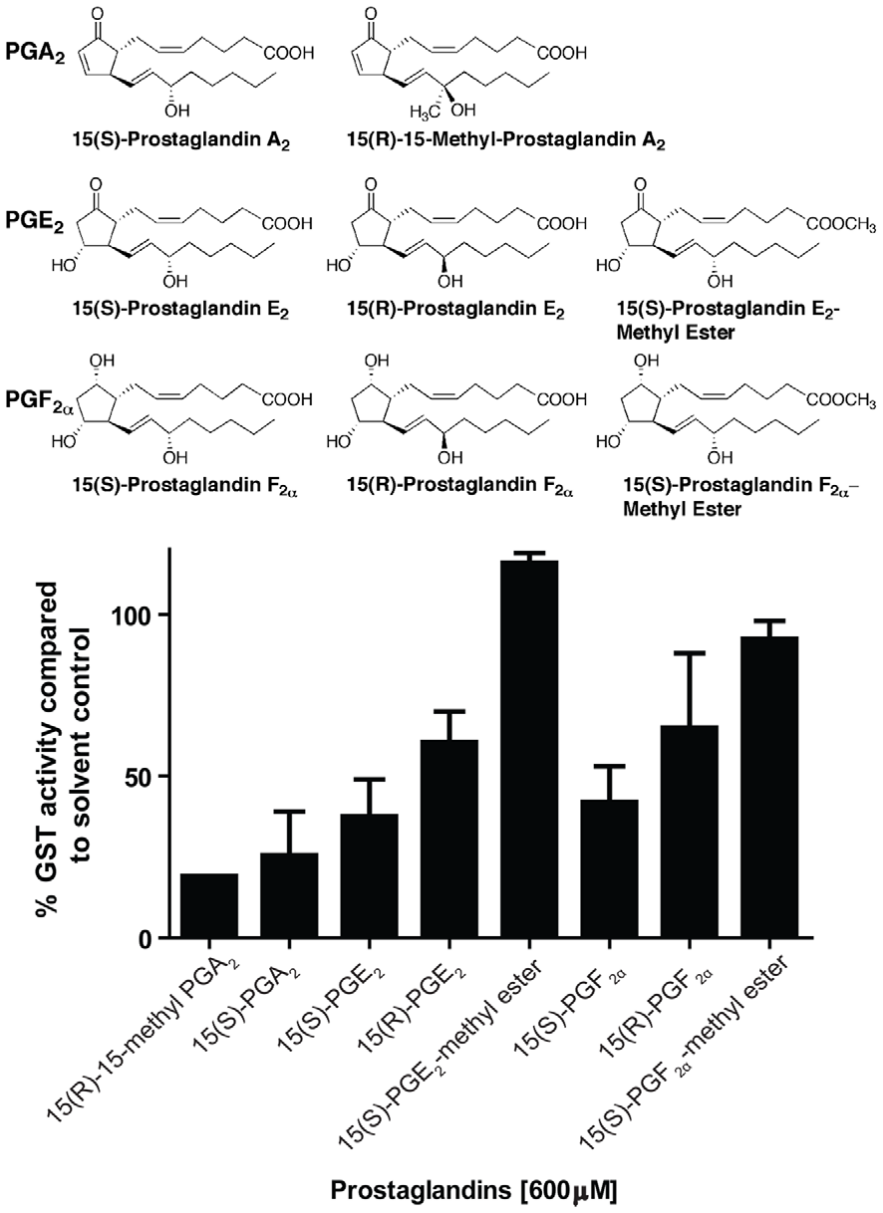
Prostaglandin A, E and F series inhibit *Cyphoma* GST activity. Eight commercially available prostaglandin compounds, representing a diversity of prostaglandin series present in gorgonian tissues, were screened for their ability to inhibit GST activity at 600 µM. Bars represent the mean (±SE) percent GST activity remaining after prostaglandin exposure compared to solvent controls. The reaction mixture consisted of 2 µg of cytosolic GST protein in 0.1 M potassium phosphate buffer, pH 7.5, containing 1 mM GSH, 1 mM CDNB at 25°C with 5% (v/v) DMSO. Digestive gland cytosolic preparations from two snails were used as the enzyme source with specific activities of 5.8 and 5.1 µmol min^−1^ mg protein^−1^, respectively.

**Figure 4:**
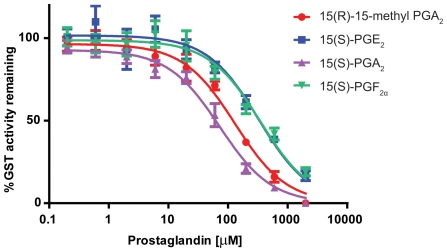
Inhibition of *Cyphoma* affinity-purified GST activity by select prostaglandins. Points represent the mean (±SE) GST activity remaining compared to solvent controls after incubation with various concentrations (0.2–2000 µM) of 15(R)-15-methyl PGA_2_ (•), 15(S)-PGE_2_ (▪), 15(S)-PGA_2_ (▴), 15(S)-PGF_2α_ (▾). The reaction mixture consisted of 3.3–6.4 ng of affinity-purified GST protein in 0.1 M potassium phosphate buffer, pH 7.5, containing 1 mM GSH, 1 mM CDNB at 25°C with 5% (v/v) DMSO. Affinity-purified GST preparations from five snails were used as the enzyme source with specific activities of ranging from 290–582 µmol min^−1^ mg protein^−1^. Plots were used to obtain the IC_50_ value for each prostaglandin and are listed in Table 1.

When commercially available prostaglandins representing a diversity of classes found in *P. homomalla* were screened, those compounds containing a cyclopentenone ring (e.g., PGA_2_) caused the greatest inhibition of GST activity, whereas the methyl ester forms of PGE_2_ and PGF_2α_ displayed little to no inhibitory activity in comparison to solvent controls (Figure 3). The potencies of the four most inhibitory prostaglandins (15(S)-PGA_2_, 15(R)-15-methyl PGA_2_, 15(S)-PGE_2_, 15(S)-PGF_2α_) were further evaluated at a range of concentrations (0.2–2000 µM) (Figure 4). All prostaglandins displayed concentration-dependent inhibition of enzyme activities, with IC_50_ values ranging from 75.4 µM for 15(S)-PGA_2_ to 334.6 µM for 15(S)-PGF_2α_ (Table 1). Those prostaglandin series with the greatest inhibitory potencies (e.g., PGA_2_) are known to be in the highest abundance in gorgonian tissues [28]–[31]. The K_i_ values for 15(S)-PGA_2_, 15(R)-15-methyl PGA_2_, 15(S)-PGE_2_, and 15(S)-PGF_2α_ calculated using the Cheng-Prusoff equation, ranged from 21.7 to 96.4 µM (Table 1).

**Table 1:** IC_50_ values and inhibition constants (K_i_) of prostaglandin inhibitors.

Inhibitor	IC_50_ value (µM)^a^	R^2^	K_i_ (µM)^b^
15(S)-PGA_2_	75.4	0.946	21.7^c^
15(R)-15-methyl PGA_2_	136.7	0.930	39.4
15(S)-PGE_2_	312.2	0.821	90.0
15(S)-PGF_2α_	334.6	0.848	96.4

a Enzyme activity in the presence of prostaglandin inhibitors was determined at pH 7.5 and 25°C, with 1 mM CDNB and 1 mM GSH and 4% (v/v) DMSO. The mean percent inhibition from five affinity-purified GST concentrates was used to calculate IC_50_ values.

b K_i_ values were calculated using the Cheng-Prusoff equation with an K_m_ value of 0.41 mM for CDNB obtained from the average of two independent experiments; a CDNB substrate concentration of 1 mM; and IC_50_ values listed in the table above.

c In an attempt to constrain the likely range of calculated K_i_ values, test calculations using K_m_ values for CDNB ranging from 0.1 mM to 1.0 mM resulted in K_i_ values for 15(*S*)-PGA_2_ of 6.0 to 37.7 µM.

## Discussion

The exploitation of allelochemically-defended gorgonian corals by the co-evolved predator, *Cyphoma gibbosum*, is likely to be facilitated by this predator's ability to biotransform and/or sequester dietary allelochemicals using detoxification enzymes, such as soluble glutathione *S*-transferases. GSTs are integral components of the cellular xenobiotic defense system [47] and have been documented to mediate allelochemical tolerance in terrestrial consumers [4]–[6]. Our results suggest that they may also be important for marine predators that consume chemically defended prey; *C. gibbosum*'s high, constitutive expression of GSTs may protect this consumer from the abundance of deterrent lipophilic compounds found in its gorgonian diet.

In a controlled feeding assay we determined that digestive gland tissues from *C. gibbosum* constitutively express high levels of GST activity regardless of the gorgonian diet. This finding differs from that of a previous study [14] that noted differences in GST activity from field-collected *C. gibbosum* from different gorgonian hosts. The apparent differences could reflect differences in experimental design between the two studies. Vrolijk & Targett (1992) noted differences in GST activity among field-collected individuals for which no data were available on the residence time of snails on their respective hosts. In contrast, snails in the present study were subject to controlled, four-day feeding assays. It is possible that GST enzymes could show significant levels of induction if snails were allowed to feed on gorgonian diets longer than four days. However, studies of GST induction in other invertebrates [4], [48] suggest that four days is sufficient for induction to occur; thus, it seems unlikely that our design, which included those gorgonian species examined by Vrolijk & Targett (1992), would have missed significant induction of GSTs. Snails could conceivably extend their exposure to the same suite of allelochemicals beyond the average 3.3 day residence time [27] migrating to another colony of the same species. This scenario would be favored if *B. asbestinum* and *G. ventalina*, the two gorgonian diets eliciting increased GST activity in Vrolijk & Targett (1992), were in higher abundance on reefs, because prey selection by *C. gibbosum* is in proportion to gorgonian species abundance [27]. Alternatively, geographical [23] or within colony differences in allelochemical content [49] could account for the differences between the two studies.

Although GST activity did not vary by gorgonian diet, cytosolic digestive gland GSTs were further purified by affinity chromatography to investigate if GST subunit composition was influenced by allelochemical exposure. *Cyphoma* GST subunits were separated by HPLC, resulting in the identification of two major mu-class GST subunits, which accounted for 93% of the total GST subunit abundance. Quantification of GST subunit composition indicated that the relative abundance of GST subunits did not differ among snails feeding on different gorgonian diets. Interestingly, GST activity was maintained at a high level and subunit composition did not vary in snails fed control diets devoid of allelochemicals as compared to snails fed gorgonian diets. The presence of high GST activity in control-fed snails could indicate that some lipophilic gorgonian compounds and/or their metabolites may persist in snail tissues even after feeding has ceased, causing the expression of GSTs to be maintained. Alternatively, the constitutive expression of *Cyphoma* GSTs could be regulated by an allelochemical-independent mechanism. In either case, having a constant supply of ‘all-purpose’ GST enzymes may prove advantageous for predators that consistently feed on prey containing allelochemical GST substrates.

The majority of GST substrates are hydrophobic compounds that react with the thiol moiety of glutathione [2]. In our bio-assay guided screening approach, we used the ability of extracts/compounds to inhibit GST activity as an indirect measure of their potential to act as GST substrates. The results of GST inhibition assays indicated that the chloroform-soluble fractions from gorgonian extracts contained the bulk of inhibitory compounds. However, in addition to containing potential GST substrates, gorgonian extracts may also contain electrophilic compounds that could act as potent GST inhibitors, binding to free cysteine residues on the protein resulting in enzyme inactivation [50]. The presence of high affinity GST inhibitors in gorgonian tissues may represent specific counter-adaptations of prey to thwart consumer GST-mediated metabolism of co-occurring allelochemicals [3], [21]. Although our initial screening approach of gorgonian extracts was not able to distinguish between GST substrates and inhibitors, this result did substantiate the hypothesis that all gorgonian species contained significant quantities of compounds capable of interacting with *Cyphoma* GSTs, which could account for the high constitutive activity of digestive gland GSTs identified here.

The gorgonian *Plexaura homomalla* is a favored diet of *C. gibbosum* [26], [27], [45], despite having high tissue concentrations of deterrent cyclopentenone prostaglandins. Electrophilic eicosanoids, like the cyclopentenone prostaglandin PGA_2_, have been recognized as high affinity substrates/inhibitors for vertebrate GSTs [39]–[41], [50]–[53]. Furthermore, NMR and LC-MS analysis showed that PGA_2_ was present in the *P. homomalla* HPLC fractions demonstrating the greatest inhibitory potential. Therefore, the potential importance of *P. homomalla*'s allelochemicals in the co-evolution of *C. gibbosum* detoxification enzymes, coupled with the interesting ecological and biological activities of eicosanoids, prompted us to determine if prostaglandins could serve as substrates for *C. gibbosum* GSTs.

*P. homomalla* tissues predominately contain the fully esterified form of PGA_2_ (∼2% dry weight of the gorgonian) [30], which is related to a larger group of eicosanoids that includes the coral-derived halogenated marine clavulones [54], [55] and puniglandins [56], [57], all of which display cytotoxic activities thought to be related to the presence of a reactive α,β-unsaturated ketone [58], [59]. While the exact mechanism of toxicity is unknown, the prostaglandins are transported into the nucleus [60]–[62] where the electrophilic α,β-unsaturated carbonyl is free to bind with nucleophilic sulfhydryl residues on target proteins, unless rapidly conjugated by cytosolic GSH and transported out of the cell by glutathione-conjugate transporters [63]. In this study, the α,β-unsaturated carbonyl-containing prostaglandins (15(R)-15-methyl PGA_2_ and 15(S)-PGA_2_) were the most potent inhibitors of CDNB-conjugating activity of *Cyphoma* GSTs in both the initial screening of eight prostaglandin compounds and upon comparison of IC_50_ values, establishing the order of potency of prostaglandins to be 15(S)-PGA_2_>15(R)-15-methyl PGA_2_≫15(S)-PGE_2_≈15(S)-PGF_2α_. The K_i_ values for cyclopentenone-containing prostaglandin A series were also 2.3- to 4-fold lower (greater affinities) for *Cyphoma* GSTs in comparison to those of either PGE_2_ or PGF_2α_.

If we assume PGA_2_ is a substrate for *Cyphoma* GST(s), possibly binding with high affinity in the active site (H-site) once occupied by CDNB, it is reasonable to compare K_i_ values (i.e., apparent K_m_ for PGA_2_) obtained here to K_m_ values for PGA_2_ cited in other studies. The apparent K_m_ (∼21.7 µM) for 15(S)-PGA_2_ described here is in line with values identified for the conversion of PGA_2_ to its glutathione conjugate by human mu-class GST M1a-a (26 µM) [37], rat alpha-class GST A4-4 (12 µM) and human mu-class GST M2-2 (7.6 µM) [64]. The rank order of GST affinity for prostaglandins (15(S)-PGA_2_>15(R)-15-methyl PGA_2_>15(S)-PGE_2_>15(S)-PGF_2α_), is also positively correlated with the relative abundance of each prostaglandin series in *P. homomalla* tissues. This finding may suggest that *Cyphoma* GSTs have evolved to efficiently catalyze the conjugation of prostaglandins found in the greatest abundance in its diet (PGA_2_), yet still retain a broad enough substrate specificity to metabolize additional prostaglandin classes (PGE_2_, PGF_2α_).

The apparent K_m_ (Ki) values reported here indicate that dietary prostaglandins could be high affinity substrates for *Cyphoma* GSTs *in vivo*. However, the physiological relevance of this value would depend on the concentration of prostaglandins occurring in digestive gland tissues of *Cyphoma*. To obtain an estimate of these concentrations, we first calculated the volume of *P. homomalla* tissue consumed per snail per day based on feeding scar measurements [27]. *Cyphoma* feeding scars on *P. homomalla* colonies averaged 12 cm in length, did not exceed 1 cm in width, and penetrated to the gorgonian skeleton 66% of the time [27], a depth of 0.4 cm (K. Whalen, pers. observation). Therefore, conservative estimates of scar volume averaged 1.44 cm^3^ per snail or 1.44 mL of gorgonian tissue. This tissue volume was divided by the mean residence time of snails feeding on *P. homomalla* (2.9 d, n = 50 snails) [27] to yield an estimate for the volume of *P. homomalla* tissue consumed by each snail per day (0.66 mL/snail/day). Pawlik & Fenical [34] determined that 1 mL of wet *P. homomalla* tissue (excluding the gorgonian axial skeleton) was equivalent to 0.86 g of dry gorgonian tissue. If 2% of the dry weight of the gorgonian is prostaglandins [30], then 0.66 mL of gorgonian tissue would contain 0.011 g of prostaglandins. Assuming the majority of prostaglandins are in the PGA_2_ form (FW = 348.5 g) and are completely retained within the digestive gland upon ingestion (ave. dig. gland weight = 0.25 g, K. Whalen pers. obs., with a density comparable to human liver ∼1 g/mL), then the upper limit of PGA_2_ concentrations in digestive gland would be 0.13 M. The single day grazing rates of *P. homomalla* colonies by *C. gibbosum* of 0.17 mL/snail/day reported in [26] were used to obtain the lower bound of PGA_2_ tissue concentration (0.03 M). Ciereszko and Schneider [65] reported the fecal pellets of *C. gibbosum* contain no appreciable amounts of recognizable prostaglandins, suggesting that the majority of prostaglandins are being metabolized or sequestered within the snail. If we conservatively assume that only 1% of the ingested prostaglandins are retained within the digestive gland during feeding (e.g., 1% concentration of PGA_2_∼0.3–1.3 mM PGA_2_), the *in vivo* concentration of prostaglandins in this tissue would still be 7- to 59-fold higher than the apparent K_m_ (21.7–39.4 µM) obtained for PGA_2_ and its methylated derivative (Table 1). Even at the lower bound of *in vivo* PGA_2_ concentration (∼0.3 mM), *Cyphoma* GSTs would be operating at near physiological capacity (>93%) according to the fractional velocity (v/V_max_) estimates.

Glutathione *S*-transferases are most well known for their ability to conjugate electrophilic toxicants; however, their capacity to bind and sequester non-substrate ligands may also be an important protective mechanism [66], [67]. Certain human GST isoforms have been shown to exert their protective effects through this ligandin-like behavior by binding with high affinity to inhibitory prostaglandins (e.g., PGJ_2_), effectively sequestering them in the cytosol away from target nuclear proteins and preventing effects on gene regulation [39], [43]. A comprehensive screening of allelochemicals from host plants of the fall armyworm *Spodoptera frugiperda* found that many act as non-competitive inhibitors of GST activity [5]. Overexpression of GSTs in *S. frugiperda* may serve as a detoxification strategy by facilitating the sequestration of non-substrate ligands and thereby preventing their interference with essential cellular functions. The same strategy might be used by the marine chiton *Cryptochiton stelleri*, which consumes a red algal diet rich in the feeding deterrent lanosol, a noncompetitive inhibitor of this chiton's GST activity [13]. Similarly, high constitutive GST activity was observed in *Cyphoma* independent of allelochemical diet and all of the gorgonian extracts examined contained potent inhibitory compounds. While the type of inhibition was not quantified for gorgonian extracts and all compounds, it is likely that gorgonian diets contain both substrates (e.g., PGA_2_) and non-substrate ligands. Therefore, the constitutive expression of GSTs may be indicative of a more *general* biochemical resistance strategy that is capable of responding to a diversity of compounds in the diet of a generalist consumer.

The results of this study provide the first comprehensive evaluation of the influence of dietary allelochemicals on the expression and function of glutathione transferases in a generalist marine consumer. Together with our companion studies on the gorgonian diet-mediated expression of cytochrome P450 expression in *Cyphoma* [68], the present results add substantial knowledge regarding the role of detoxification enzymes in determining macroevolutionary patterns of diet preference among consumers. Controlled feeding assays showed that *Cyphoma* digestive gland GST composition and activity did not differ with gorgonian diet. This result in combination with evidence from *in vitro* inhibition studies with *Cyphoma* GSTs by gorgonian extracts, suggests that the high constitutive expression of GST enzymes in *Cyphoma* digestive gland may be necessitated by the presence of numerous potent inhibitors/substrates in their gorgonian diets. Furthermore, all three prostaglandin classes (A, E, F) found in the gorgonian *P. homomalla* were able to inhibit *Cyphoma* CDNB-conjugating GST activity, with relative potencies positively correlated with their abundance in gorgonian tissues. Together, these findings suggest that *C. gibbosum* detoxification enzymes may have evolved to enable the conjugation and sequestration of a broad range of lipophilic allelochemicals resulting from this predator's close association with chemically diverse gorgonian diets. Given the importance of allelochemicals in shaping patterns of predation and herbivory in marine systems, these findings suggest that co-evolved consumers have the capacity to detoxify allelochemicals in their prey, providing these consumers with a competitive advantage in ecosystems where allelochemically-rich prey species abound.

## Methods

### Materials

CDNB, DTT, potassium phosphate, potassium chloride, EDTA, protease inhibitor cocktail (4-(2-aminoethyl)benzenesulfonyl fluoride, aprotinin, bestatin hydrochloride, E-64, leupeptin, and pepstatin A), GSH-agarose (G4510) was purchased from Sigma (St. Louis, MO). Bradford reagents were purchased from Bio-Rad (Hercules, CA). PD-10 size exclusion columns were purchased from GE Healthcare (Piscataway, NJ). Amicon Ultra-4 centrifugational filters were purchased from Millipore (Billerica, MA). NanoOrange protein quantitation kit was purchased from Molecular Probes (Eugene, OR). Prostaglandins (15(S)-PGA_2_, 15(R)-15-methyl PGA_2_, 15(S)-PGE_2_, 15(R)-PGE_2_, 15(S)-PGE_2_-methyl ester, 15(S)-PGF_2α_, 15(R)-PGF_2α_, 15(S)-PGF_2α_-methyl ester) were purchased from Caymen Chemical (Ann Arbor, MI). All solvents used for extract and chemical analysis were purchased from Fisher Scientific (Pittsburgh, PA).

### Animal Collection and Feeding Assay Design

A total of 39 adult *Cyphoma gibbosum* (ca. 2–3 cm length) were collected from four shallow reefs (<20m) (Big Point – 23°47.383′N, 76°8.113′W; Rainbow Gardens – 23°47.792′N, 76°8.787′W; Shark Rock – 23°45.075′N, 76°7.475′W; Sugar Blue Holes – 23°41.910′N, 76°0.23′W) surrounding the Perry Institute of Marine Science (PIMS), Lee Stocking Island, Exuma Cays, Bahamas in January 2006. Snails were immediately transported to wet laboratory facilities provided by PIMS where a series of feeding assays were conducted with six gorgonian species (*Briareum asbestinum*, *Eunicea mammosa*, *Gorgonia ventalina*, *Pseudopterogorgia acerosa*, *Pseudopterogorgia americana*, *Plexaura homomalla*) observed to serve as hosts for *C. gibbosum* in the field.

Individual snails were housed separately in 3-L polycarbonate tanks which were placed in a 12′×20″ raceway supplied with filtered, continuous-flow seawater at a flow rate of approximately 1L min^−1^. This design allowed for a common water source to feed each tank but prevented mixing between tanks. Snails collected from the same reefs were housed separately in the same raceways and randomly assigned to one of seven groups – one of six gorgonian diets or a control diet – at the start of the feeding assays. Snails were allowed to feed ad. libum on either a control diet devoid of gorgonian compounds (i.e., alginic acid and freeze-dried squid) (n = 12) or one of six gorgonian diets (n = 27) for four days.

A minimum of ten colonies for each gorgonian species were collected from shallow reefs (<20m) surrounding PIMS and housed in a separate raceway prior to introduction into the tanks containing *C. gibbosum*. The maximum amount of time between gorgonian field collection and introduction into the feeding assay was 12 hours. Gorgonian colonies were cut into 2–3 inch pieces and allowed to recover for four hours before addition to *C. gibbosum* tanks. The control diet consisted of alginic acid and freeze-dried squid powder prepared as described in [20] and mirrored the average nutritional quality of gorgonian tissue. The squid-alginate paste was pressed into sixteen 3-mm deep wells drilled into a 3″×1″ piece of Formica® resembling a domino. The domino was then placed into a 0.25 M calcium chloride solution allowing the squid-alginate paste to harden. Both control and gorgonian diets were replaced every 24 hours for four days and feeding activity was monitored by the presence of feeding scars on their gorgonian prey and empty wells on control dominos. Following the completion of the feeding assay digestive glands were immediately dissected, weighed, frozen in liquid nitrogen and maintained at −80°C until further processing.

### GST Purification and HPLC Subunit Analysis

Cytosolic and affinity-purified GSTs were isolated from *Cyphoma* digestive gland samples as described in [44]. Briefly, cytosolic GSTs were isolated by homogenizing digestive glands (n = 39) separately in buffer (0.1 M potassium phosphate, 1 mM EDTA, 1 mM DTT, 1.15% potassium chloride, protease inhibitor cocktail (1×); pH 7.5), and differentially centrifuging the homogenates to obtain the cytosolic fraction containing the soluble GST pool. Individual cytosolic GST fractions were then applied to both a PD-10 size exclusion and GSH-agarose affinity column in series to obtain the affinity-purified fraction of GSTs. GST fractions were buffer exchanged to low salt concentration and concentrated with Amicon Ultra-4 centrifugational filters (5K NMWL membrane) and protein concentrations of Amicon concentrates were determined using the NanoOrange protein quantitation kit.

A 30 µL aliquot of the affinity-purified GST concentrate from each of the 39 digestive gland samples was injected onto a reverse phase Vydac protein/peptide column (model #218 TP 52; C18 µm 250 mm×2.1 mm) and separated using a Waters 600 MultiSolvent Delivery System, with a flow rate of 0.5 mL/min. Peaks were detected using a Waters 2487 Dual Wavelength Absorbance Detector (λ = 214 nm). Mobile phase A consisted of 38% acetonitrile, 62% water and 0.1% trifluoroacetic acid (TFA). Mobile phase B consisted of 80% acetonitrile, 20% water and 0.1% TFA. The initial mobile phase consisted of 100% A. GST subunits were separated using a linear gradient from 0 to 40% B in 22 min, and 40 to 100% B in 37 min. The column was re-equilibrated with 100% A from 37–50 mins prior to the next injection. Integration of HPLC peak area was achieved using the Empower 2 Chromatography Data Software package (Waters, Milford, MA) and converted to GST subunit percent composition for each digestive gland sample.

### GST Activity Assay

Enzyme activity was measured using CDNB as a substrate by the method of [69] optimized for *C. gibbosum* [14], [44] in a microplate format. The reaction mixture (in a final volume of 200 µL) contained 0.1 M potassium phosphate buffer, 1.0 mM EDTA, pH 7.5, 1 mM CDNB, 1 mM reduced GSH and 2 µg of cytosolic protein or 3.3–6.4 ng of affinity-purified GST sample. CDNB was solubilized in ethanol and constituted 1% of the final reaction mixture volume. The reaction incubated at 25°C was initiated by the addition of CDNB and performed in triplicate. The conjugation of CDNB with GSH was measured as the increase in absorbance at 340 nm (Δε_340_ 0.00503 µM^−1^ cm^−1^) using a tunable microplate reader (Versamax, Molecular Devices, Sunnyvale, CA). Activity was calculated using protein concentrations determined via the Bradford assay with BSA as a standard.

### Extraction and Isolation of Gorgonian Compounds

A minimum of ten colonies for each gorgonian species were collected from shallow reefs (<20 m) surrounding Lee Stocking Island, Exuma Cays, Bahamas. A portion of the gorgonian colonies, prior to their introduction into the feeding assay, was immediately removed after field collection and immersed in seawater to determine volumetric displacement, frozen at −80°C, and lyophilized for subsequent chemical extraction. *Pseudopterogoria blanquillensis* was also collected for chemical analysis; however, this gorgonian species did not participate in the feeding assays. A 50 mL volumetric equivalent of pooled tissue for each of the eight gorgonian species was extracted twice at room temperature in 250 mL reagent grade acetone overnight with agitation. Resulting crude organic extracts were vacuum-filtered through celite, dried by rotary evaporation, and recombined into a 20 mL scintillation vial using a minimum volume of solvent. The crude organic extracts were then completely dried using a vacuum concentrator. Gorgonian crude organic extracts were assayed at 5% natural concentration by volume (i.e., the extract from 0.05 mL of gorgonian was diluted into 1 mL of assay buffer) for the ability to inhibit *Cyphoma* cytosolic GST activity as described below. Those crude organic extracts that were able to inhibit ≥80% of GST activity were subjected to further fractionation using a bioassay-guided fractionation approach.

Gorgonian crude organic extracts were separated by partition between hexane and methanol-water (9∶1) followed by partition of the methanol-water fraction (adjusted to 6∶4) against chloroform. The chloroform-soluble, hexane-soluble, and aqueous (i.e., methanol/water-soluble) fractions were reduced *in vacuo* and assayed for their ability to inhibit cytosolic GST activity as described below. Based on patterns of GST enzyme inhibition, chloroform fractions from all eight gorgonian species were separated further using a reverse-phase semi-prep Zorbax SB-C18 column (5 µm, 9.4 mm×2.5 cm) attached to a Waters Breeze HPLC system (515 pump) with a Waters 2487 UV detector at 215 and 254 nm. Compounds were eluted over 33 mins at a flow rate of 3 mL/min with methanol/water (9∶1) with linear ramping to 100% methanol. HPLC fractions were collected at three minute intervals over 33 min, yielding ten fractions per gorgonian species. Each chloroform fraction was assayed for GST inhibition at 10% natural volumetric concentration. Chloroform fractions yielding 100% inhibition were further assayed at 0.5% natural volumetric concentration.

### Inhibition Assays

GST activity measurements were performed as described above. Gorgonian crude organic extracts and partitions were dissolved in the appropriate solvent (e.g., acetone, *n*-propanol, or methanol), HPLC fractions were dissolved in methanol, and prostaglandins were dissolved in DMSO. Solvent concentrations did not exceed 5% of the experimental volume and had no effect on GST activity when compared to non-solvent controls (data not shown). Immediately prior to the start of the assay, inhibitor solutions were added to the buffer/GSH mixture and homogenized to ensure equal distribution of inhibitor in all microplate wells. The data were corrected for the non-enzymatic reaction rates and the effect of the inhibitors on catalytic activity was measured by comparing the initial rate of reaction in the presence and absence of the inhibitor.

Eight commercially available prostaglandins representing a diversity of forms present in gorgonian tissue (15(S)-PGA_2_, 15(R)-15-methyl PGA_2_, 15(S)-PGE_2_, 15(R)-PGE_2_, 15(S)-PGE_2_-methyl ester, 15(S)-PGF_2α_, 15(R)-PGF_2α_, 15(S)-PGF_2α_-methyl ester), including both enantiomers (R and S) forms when possible, were screened at 600 µM for their ability to inhibit crude cytosolic GST activity. From this initial screening, only those prostaglandin compounds that demonstrated an ability to reduce GST activity by 50% or greater were further evaluated at a range of concentrations (0.2–2000 µM) in order to estimate the concentration producing 50% inhibition of enzyme activity (IC_50_). Prostaglandin IC_50_ values were calculated and 95% confidence intervals were estimated using Prism 5.0 software (GraphPad) by fitting the log transformation of the response variable by nonlinear regression to the variable slope equation (1) and constraining the bottom to zero but allowing the Hill Slope to vary. The variable slope equation is:
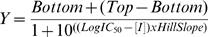
(1)where Top is the maximum percent GST activity remaining, Bottom was constrained to zero, IC_50_ is the concentration of inhibitor that produces inhibition half-way between the Top and Bottom, and [I] is the logarithmic concentration of the inhibitor.

### Determining the Dissociation Constant for Inhibitor Binding (K_i_)

Because the IC_50_ depends on the substrate concentration used in the experiment, this value is only useful for comparing inhibitors within experiments and not between laboratories unless identical assay conditions were used. However, calculated K_i_ (the dissociation constant of the enzyme-inhibitor complex) values can be used to directly compare inhibitor affinity for the enzyme between studies. K_i_ estimates were calculated using the IC_50_ values obtained for 15(S)-PGA_2_, 15(R)-15-methyl PGA_2_, 15(S)-PGE_2_, 15(S)-PGF_2α_ with the Cheng-Prusoff equation (Eq. 2), where K_m_ is the Michaelis-Menten constant for CDNB (see below), [S] is the substrate concentration (1 mM CDNB), K_i_ is the equilibrium dissociation constant for the inhibitor, and IC_50_ is as defined above.
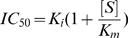
(2)To obtain an estimate of K_m_ for CDNB, initial-rate measurements using CDNB as the concentration-variable substrate were performed. GST activity was measured at five concentrations of CDNB ranging from 0.5 to 3 mM in the absence of inhibitors with 6 ng of affinity-purified GST protein at 25°C in 0.1 M potassium phosphate buffer, 1.0 mM EDTA, pH 7.5, containing 1 mM GSH and 4% (v/v) DMSO. An affinity-purified GST preparation from a single digestive gland was used as the protein source with a specific activity (mean±SE) of 561±25 µmol min^−1^ mg protein^−1^. The reaction was initiated by the addition of 2 µL of CDNB and performed in duplicate. The data from two independent experiments were corrected for the non-enzymatic reaction rates and globally fitted to the Michaelis-Menten equation to yield an estimate of K_m_ = 0.41±0.14 mM CDNB (mean±SD).

### Chemical Analysis of HPLC Fractions

Proton NMR spectra for *P. homomalla* HPLC fractions 1 and 2 were recorded in deuterated DMSO (Cambridge Isotope Laboratories, Andover, MA) with a Bruker DRX-500 instrument using a 5 mm inverse detection probe, and referenced to residual DMSO (δ 2.49 ppm). Spectra collected for chromatographic fractions were compared with the ^1^H NMR spectrum obtained for authentic 15(S)-PGA_2_ (Cayman Chemical, Ann Arbor, MI).

LC-MS analyses of *P. homomalla* HPLC fractions were completed using a Waters 2695 HPLC with a Waters 2996 photodiode array UV detector and Micromass ZQ 2000 mass spectrometer with electrospray ionization in both positive and negative modes. Optima grade solvents were used in all LC-MS experiments. LC-MS separations were achieved with an Alltech Altima C18 column (2.1×100 mm, 3 µm) applying a gradient mobile phase of 40∶60 to 95∶5 acetonitrile∶water with 0.01% acetic acid throughout. PGA_2_ was detected in fractions by matching chromatographic retention times and MS fragmentation patterns with those obtained for pure synthetic PGA_2_. For fractions in which PGA_2_ was detected, the negative-mode ESI-MS selected ion recording for *m/z* 333.3, corresponding to [M-H]^−^ of PGA_2_, was integrated and compared to a standard curve for PGA_2_ at six concentrations (r^2^ = 0.94). The concentration of PGA_2_ in each HPLC fraction was determined by interpolation of this standard curve data.

## Supporting Information

Figure S1 Representative HPLC separation of an affinity-purified extract from an individual *C. gibbosum* feeding on *B. asbestinum*. GST subunits were separated on a reverse phase VYDAC protein/peptide column (C18, 250 mm×2.1 mm) with a flow rate of 0.5 mL min-1. Mobile phase A consisted of acetonitrile/water/TFA (38∶62∶0.1, v/v/v) and mobile phase B consisted of acetonitrile/water/TFA (80∶20∶0.1, v/v/v). GST subunits were separated using a linear gradient from 0 to 40% B in 22 min, and 40 to 100% B in 37 min and visualized at 214 nm. Fourteen unique peaks were identified among all 39 digestive gland samples analyzed; however, not all were visible in one HPLC spectrum, therefore a representative spectrum was chosen. The position of HPLC peaks 1–4, 7–11, 13 and 14 are labeled.
(0.06 MB PDF)

Figure S2 Effect of snail diet on GST isoform expression.(A) Bars represent the mean (±SD; n = 39 snails) percent GST subunit composition in affinity-purified digestive gland preparations as a function of snail diet. (B) Bars represent the mean (±SD; n = 39 snails) HPLC peak area normalized for the amount of GST protein applied to the HPLC column as a function of snail diet.
(0.35 MB PDF)

Figure S3 Inhibition of *Cyphoma* GST activity by gorgonian HPLC fractions. Chloroform partitions from eight gorgonian species, (A) *B. asbestinum*; (B) *E. mammosa*; (C) *G. ventalina*; (D) *P. acerosa*; (E) *P. americana*; (F) *P. blanquillensis*; (G) *P. elisabethae*; (H) *P. homomalla*, were separated into ten fractions (indicated by dotted lines) using a reverse-phase HPLC column (Zorbax SB-C18, 9.4mm×2cm; solvent flow rate = 3 mL/min; injection volume = 500 µL). Mobile phase: methanol/water 9∶1 from 0–5 mins; linear ramping to 100% methanol from 5–18 min; 100% methanol from 18–25 min; linear gradient to initial starting conditions of methanol/water 9∶1 from 25–26 min; column flushed with methanol/water 9∶1 from 26–33 min. Absorbance was monitored at 215 and 254 nm and fractions were collected every three minutes beginning at to = 3min. Overlaid on the HPLC absorbance spectra are the results of the GST inhibition assays with affinity-purified GST protein. The reaction mixture consisted of 6 ng of affinity-purified GST protein in 0.1 M potassium phosphate buffer, pH 7.5, containing 1 mM GSH, 1 mM CDNB at 25oC with 2% (v/v) methanol. An affinity-purified GST preparation from a single digestive gland was used as the protein source with a specific activity (mean±SE) of 561±25 µmol min−1 mg protein−1. HPLC fractions were tested at 10% natural volumetric concentration (NC), unless marked by an asterisk indicating samples were further tested at 0.5% NC. Each point (▪) represents the mean of two technical replicates.
(0.32 MB PDF)

Figure S4 1H NMR spectra of (A) PGA2 standard and (B) HPLC fraction 1 from *P. homomalla*.
(0.45 MB PDF)

Figure S5 LC-MS spectra of (A) PGA2 standard and (B) HPLC fraction 1 from *P. homomalla*.
(0.34 MB PDF)

Figure S6 1H NMR spectra of (A) PGA2 standard and (B) HPLC fraction 2 from *P. homomalla*.
(0.33 MB PDF)
