# Supplementary figures and images for: Biochemical Warfare on the Reef: The Role of Glutathione Transferases in Consumer Tolerance of Dietary Prostaglandins
Source: PLoS One. 2010 Jan 6;5(1):e8537. doi: 10.1371/journal.pone.0008537 (PMC2796389; doi:10.1371/journal.pone.0008537)

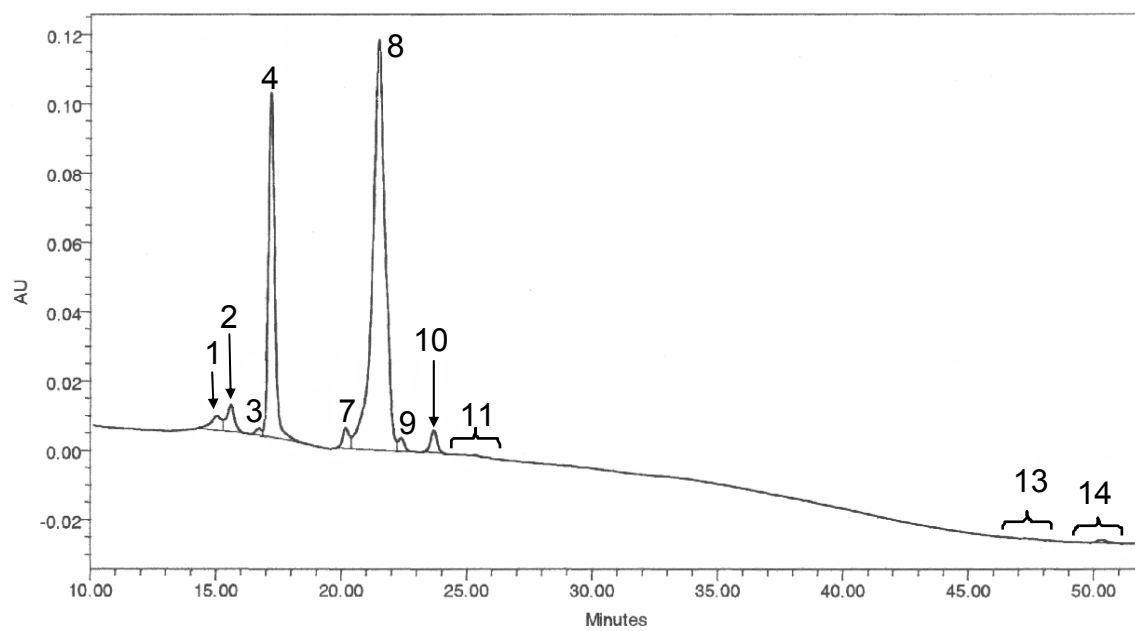

Supplement: Figure S1 — Representative HPLC separation of an affinity-purified extract from an individual C. gibbosum feeding on B. asbestinum. GST subunits were separated on a reverse phase VYDAC protein/peptide column (C18, 250 mm×2.1 mm) with a flow rate of 0.5 mL min-1. Mobile phase A consisted of acetonitrile/water/TFA (38∶62∶0.1, v/v/v) and mobile phase B consisted of acetonitrile/water/TFA (80∶20∶0.1, v/v/v). GST subunits were separated using a linear gradient from 0 to 40% B in 22 min, and 40 to 100% B in 37 min and visualized at 214 nm. Fourteen unique peaks were identified among all 39 digestive gland samples analyzed; however, not all were visible in one HPLC spectrum, therefore a representative spectrum was chosen. The position of HPLC peaks 1–4, 7–11, 13 and 14 are labeled. (0.06 MB PDF) [file pone.0008537.s001.pdf]

(A) PGA<sub>2</sub> standard

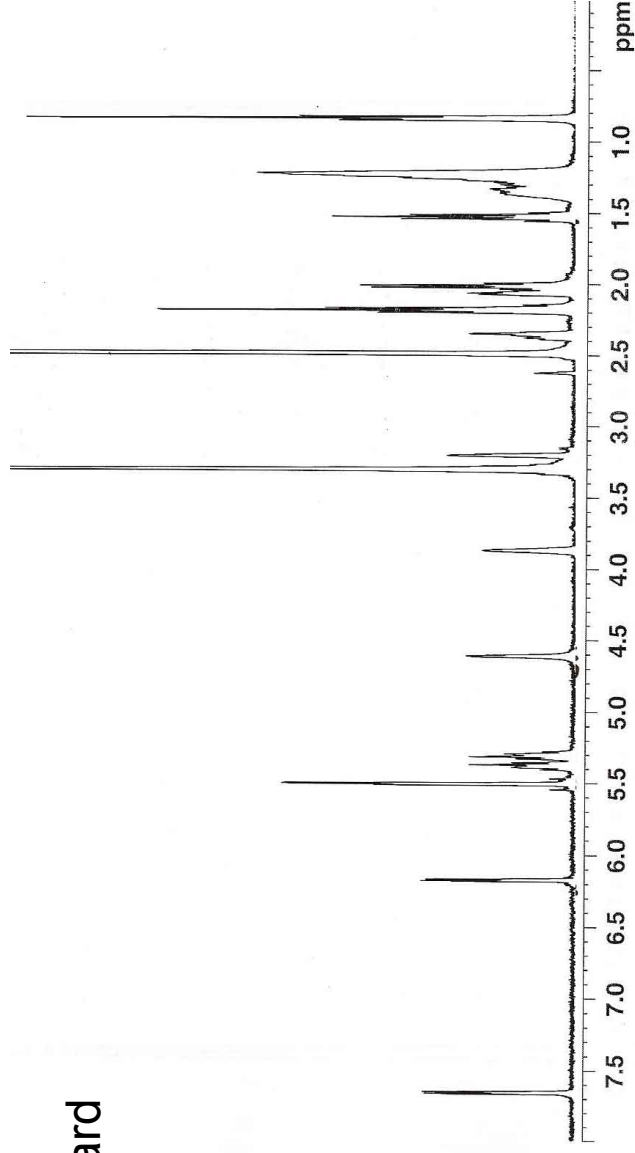

(B) HPLC fraction 1 from *P. homomalla*

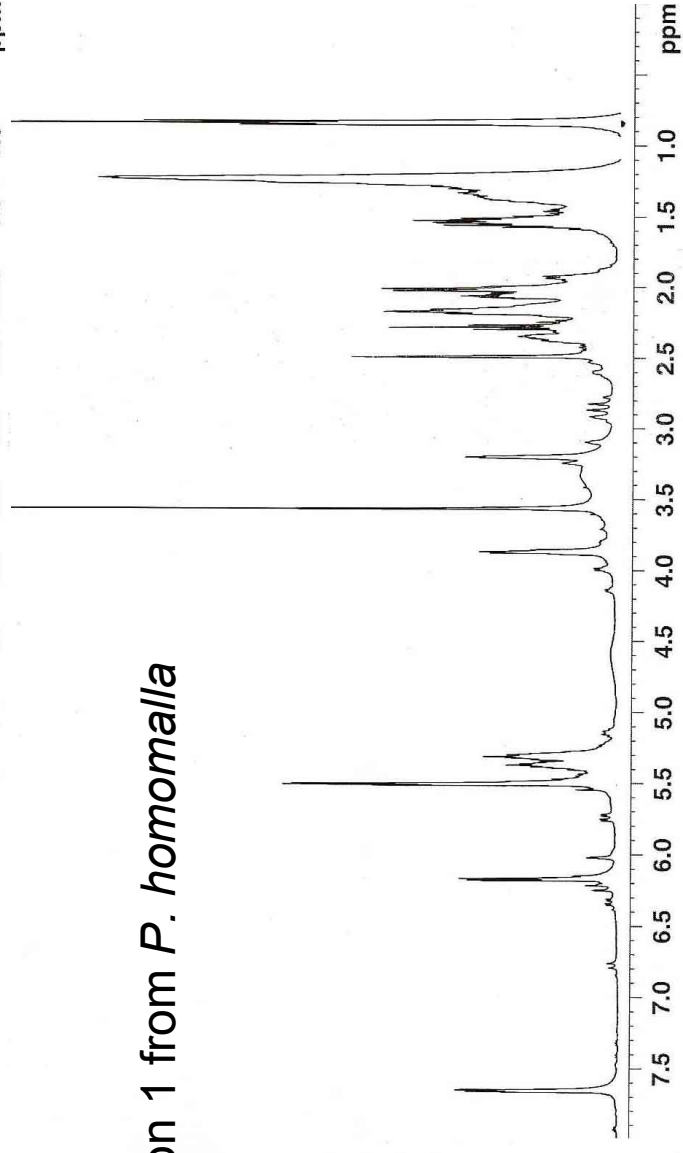

Supplement: Figure S4 — 1H NMR spectra of (A) PGA2 standard and (B) HPLC fraction 1 from P. homomalla. (0.45 MB PDF) [file pone.0008537.s004.pdf]

(A)  $\text{PGA}_2$  standard

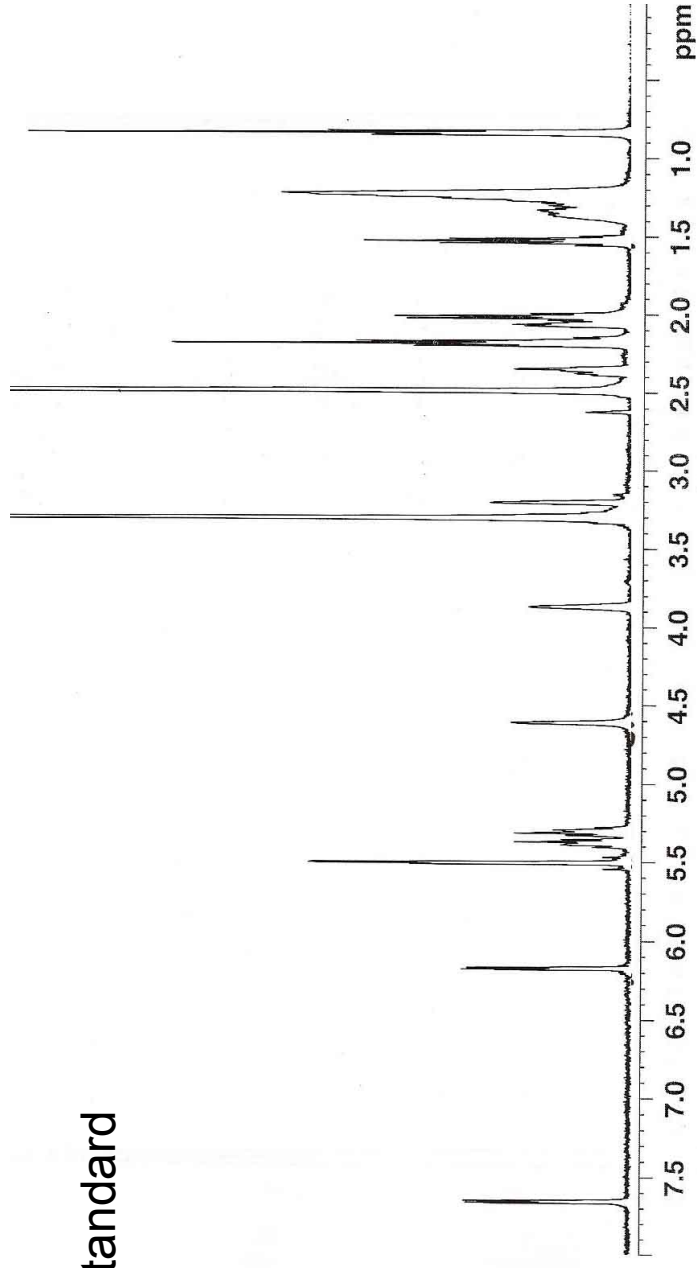

(B) HPLC fraction 2 from *P. homomalla*

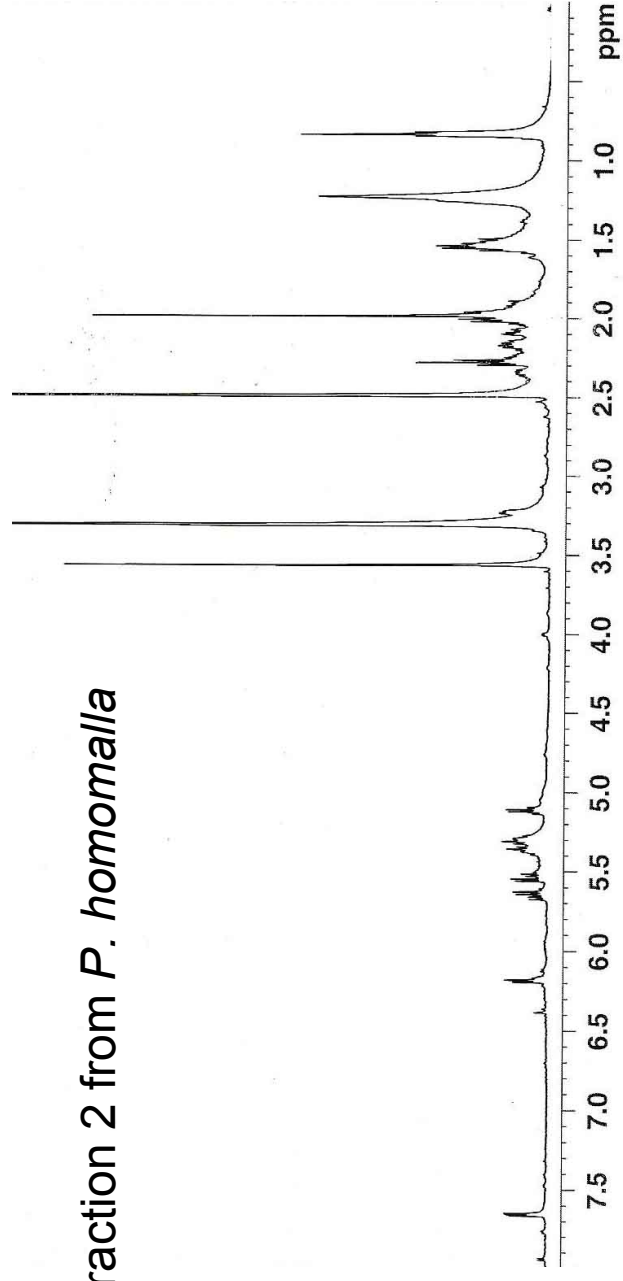

Supplement: Figure S6 — 1H NMR spectra of (A) PGA2 standard and (B) HPLC fraction 2 from P. homomalla. (0.33 MB PDF) [file pone.0008537.s006.pdf]
