# Supplementary material for: Biochemical Warfare on the Reef: The Role of Glutathione Transferases in Consumer Tolerance of Dietary Prostaglandins
Source: PLoS One. 2010 Jan 6;5(1):e8537. doi: 10.1371/journal.pone.0008537 (PMC2796389; doi:10.1371/journal.pone.0008537)
Supplement: Figure S3 — Inhibition of Cyphoma GST activity by gorgonian HPLC fractions. Chloroform partitions from eight gorgonian species, (A) B. asbestinum; (B) E. mammosa; (C) G. ventalina; (D) P. acerosa; (E) P. americana; (F) P. blanquillensis; (G) P. elisabethae; (H) P. homomalla, were separated into ten fractions (indicated by dotted lines) using a reverse-phase HPLC column (Zorbax SB-C18, 9.4mm×2cm; solvent flow rate = 3 mL/min; injection volume = 500 µL). Mobile phase: methanol/water 9∶1 from 0–5 mins; linear ramping to 100% methanol from 5–18 min; 100% methanol from 18–25 min; linear gradient to initial starting conditions of methanol/water 9∶1 from 25–26 min; column flushed with methanol/water 9∶1 from 26–33 min. Absorbance was monitored at 215 and 254 nm and fractions were collected every three minutes beginning at to = 3min. Overlaid on the HPLC absorbance spectra are the results of the GST inhibition assays with affinity-purified GST protein. The reaction mixture consisted of 6 ng of affinity-purified GST protein in 0.1 M potassium phosphate buffer, pH 7.5, containing 1 mM GSH, 1 mM CDNB at 25oC with 2% (v/v) methanol. An affinity-purified GST preparation from a single digestive gland was used as the protein source with a specific activity (mean±SE) of 561±25 µmol min−1 mg protein−1. HPLC fractions were tested at 10% natural volumetric concentration (NC), unless marked by an asterisk indicating samples were further tested at 0.5% NC. Each point (▪) represents the mean of two technical replicates. (0.32 MB PDF) [file pone.0008537.s003.pdf]

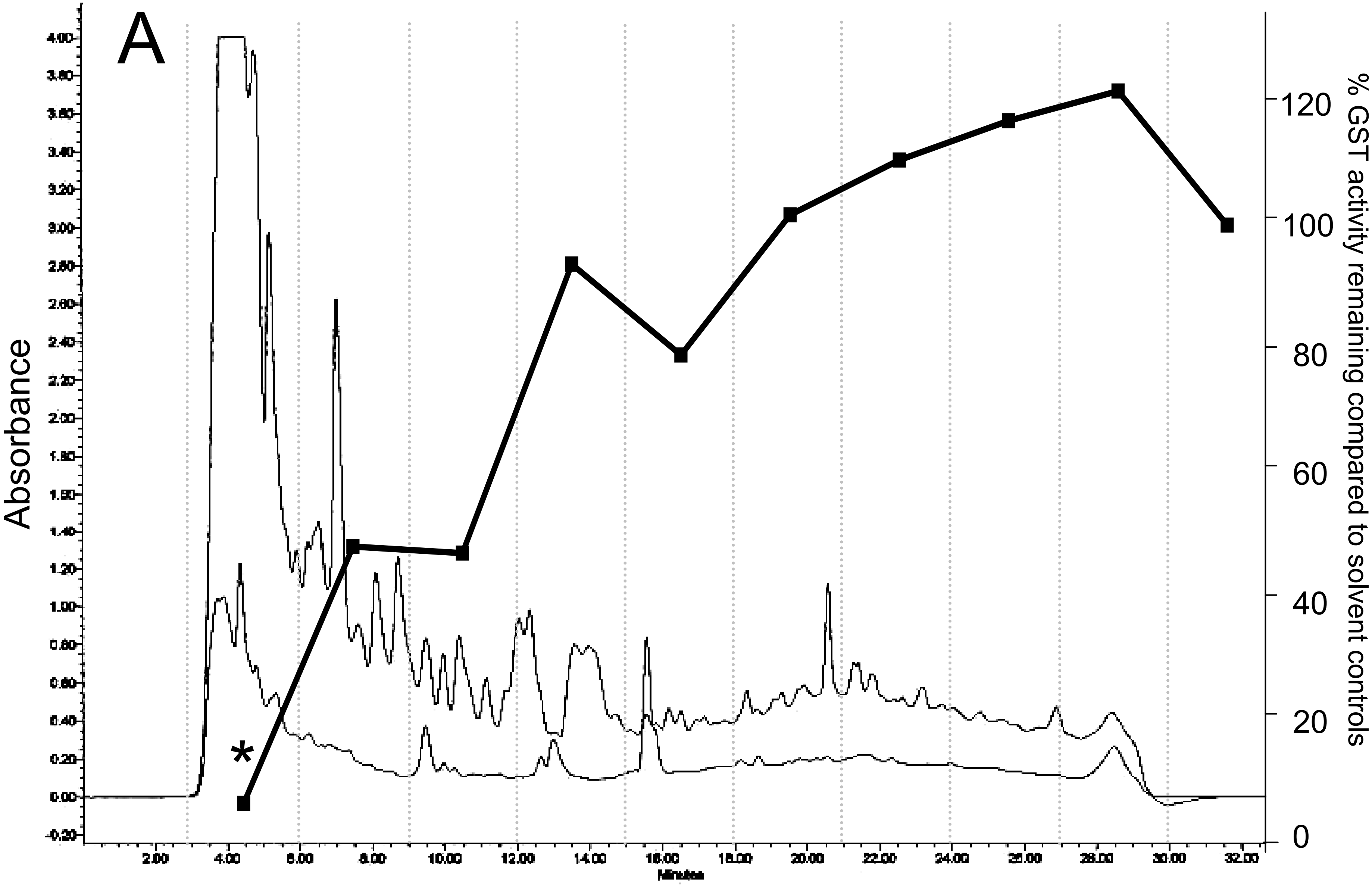

B. asbestinum

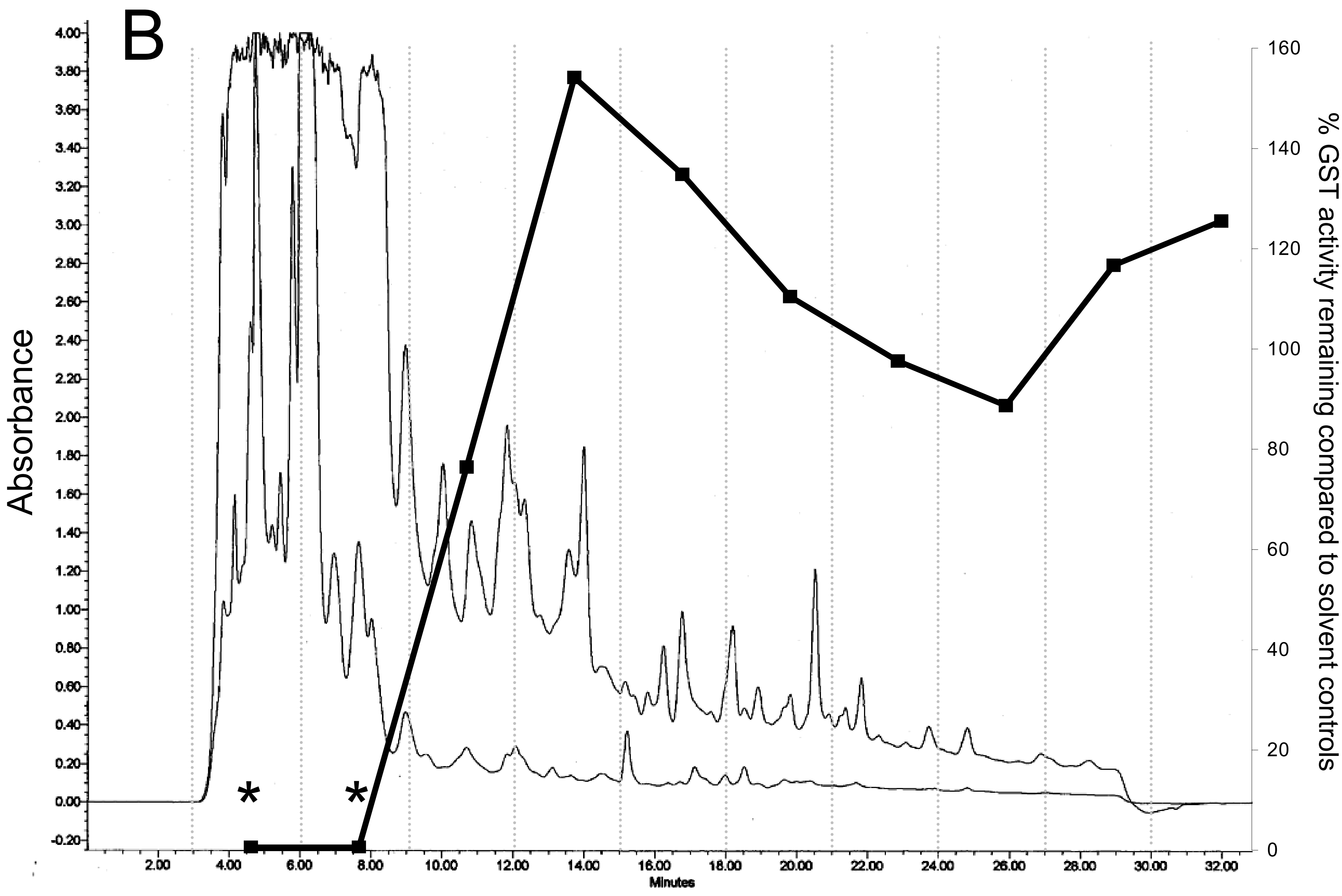
B
Absorbance
% GST activity remaining compared to solvent controls
Minutes
*
*
4.00
3.80
3.60
3.40
3.20
3.00
2.80
2.60
2.40
2.20
2.00
1.80
1.60
1.40
1.20
1.00
0.80
0.60
0.40
0.20
0.00
-0.20
2.00
4.00
6.00
8.00
10.00
12.00
14.00
16.00
18.00
20.00
22.00
24.00
26.00
28.00
30.00
32.00
160
140
120
100
80
60
40
20
0

E. mammosa

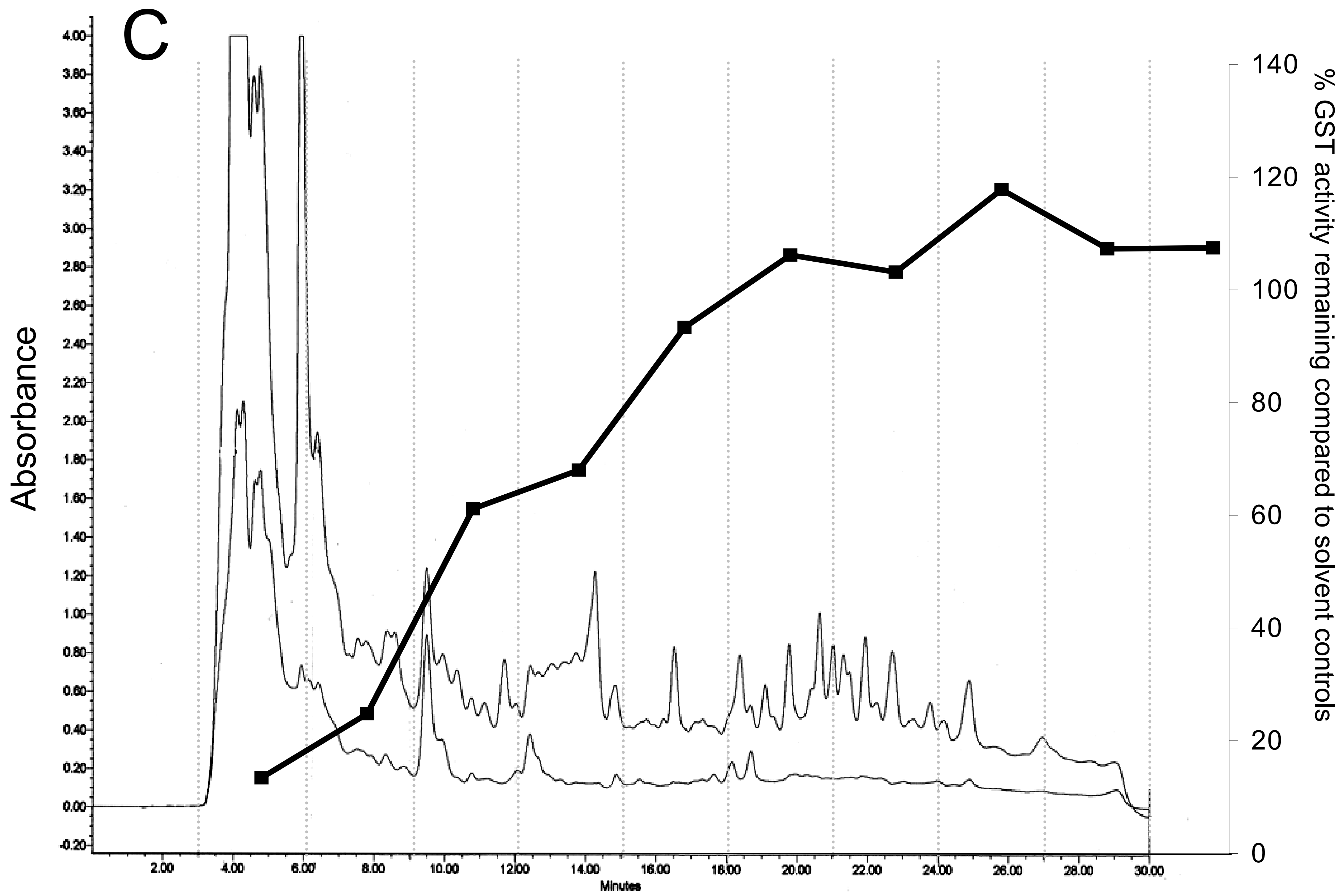
C
Absorbance
% GST activity remaining compared to solvent controls
Minutes
4.00
3.80
3.60
3.40
3.20
3.00
2.80
2.60
2.40
2.20
2.00
1.80
1.60
1.40
1.20
1.00
0.80
0.60
0.40
0.20
0.00
-0.20
2.00
4.00
6.00
8.00
10.00
12.00
14.00
16.00
18.00
20.00
22.00
24.00
26.00
28.00
30.00
140
120
100
80
60
40
20
0

G. ventalina

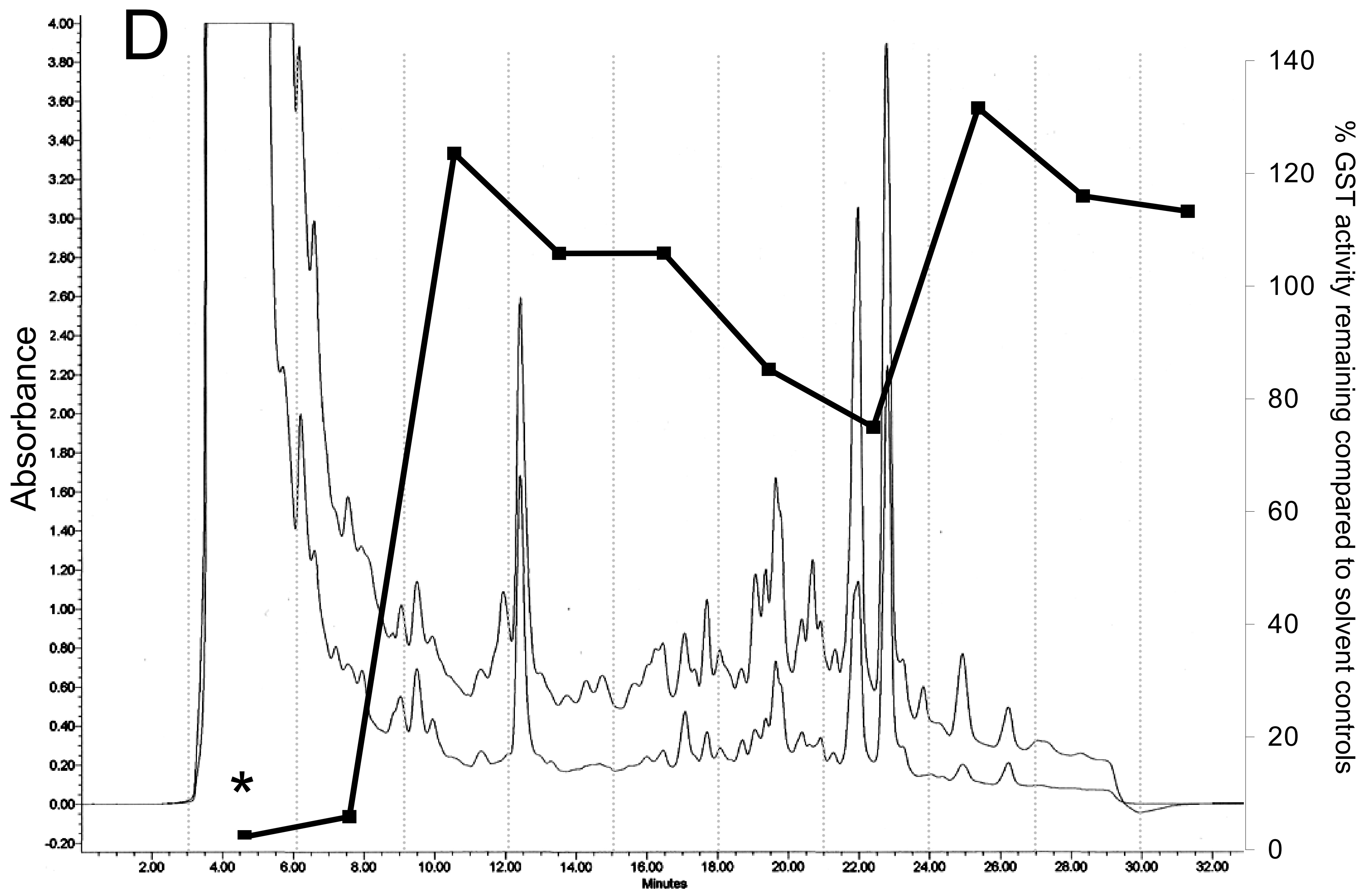
D
Absorbance
% GST activity remaining compared to solvent controls
Minutes
*
4.00
3.80
3.60
3.40
3.20
3.00
2.80
2.60
2.40
2.20
2.00
1.80
1.60
1.40
1.20
1.00
0.80
0.60
0.40
0.20
0.00
-0.20
2.00
4.00
6.00
8.00
10.00
12.00
14.00
16.00
18.00
20.00
22.00
24.00
26.00
28.00
30.00
32.00
140
120
100
80
60
40
20
0

P. acerosa

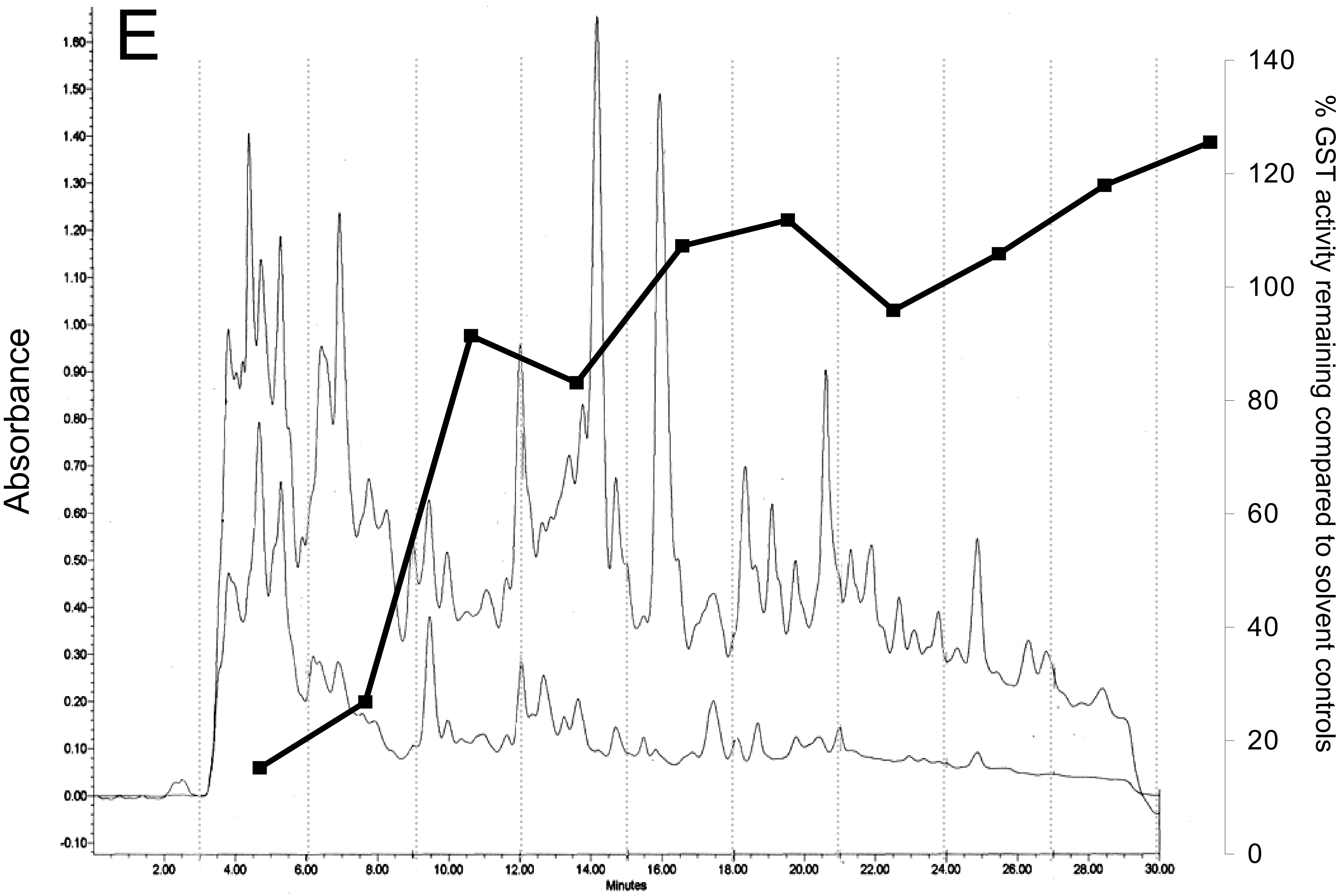

E
Absorbance
% GST activity remaining compared to solvent controls
Minutes
1.60
1.50
1.40
1.30
1.20
1.10
1.00
0.90
0.80
0.70
0.60
0.50
0.40
0.30
0.20
0.10
0.00
-0.10
2.00
4.00
6.00
8.00
10.00
12.00
14.00
16.00
18.00
20.00
22.00
24.00
26.00
28.00
30.00
140
120
100
80
60
40
20
0

P. americana

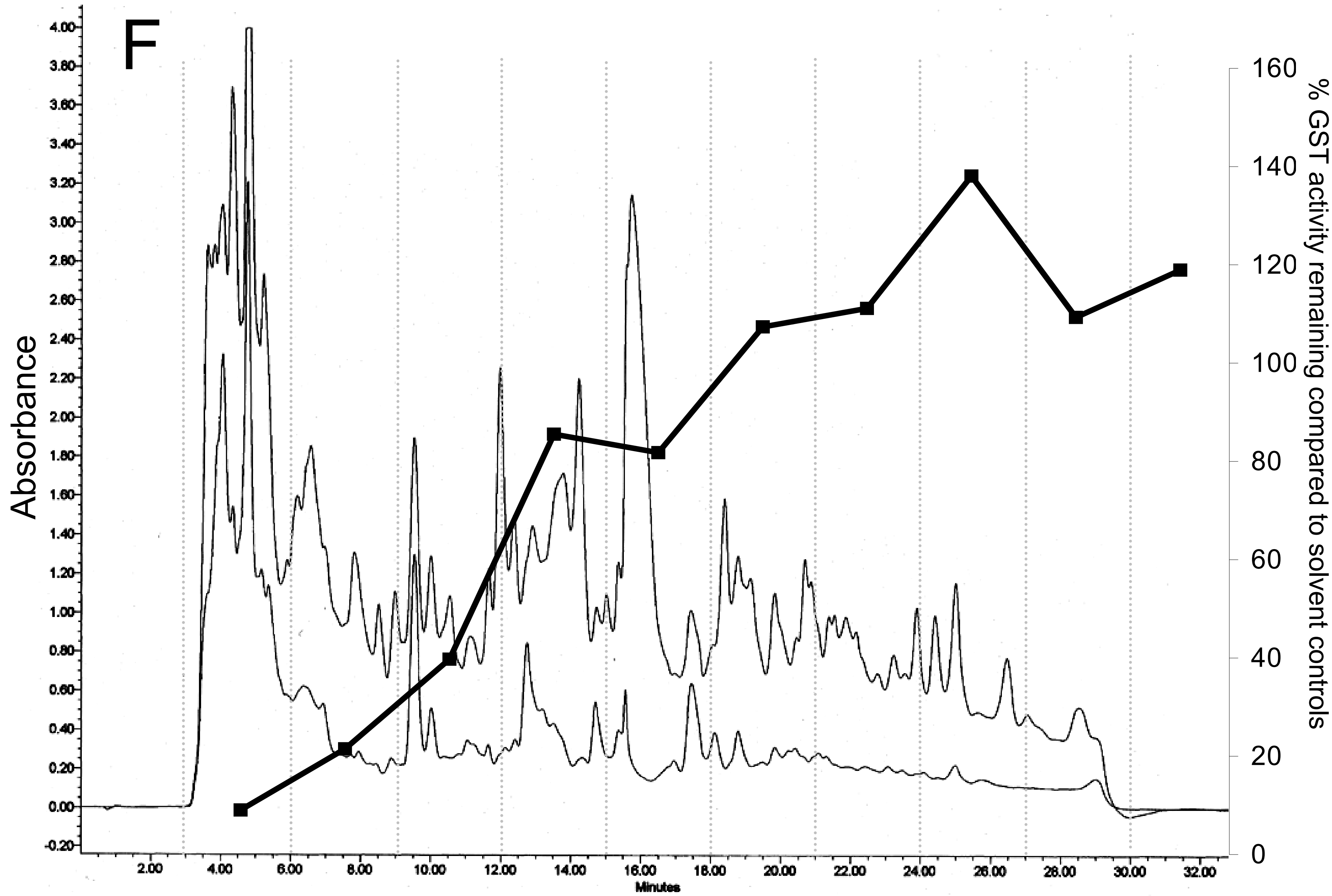

P. blanquillensis

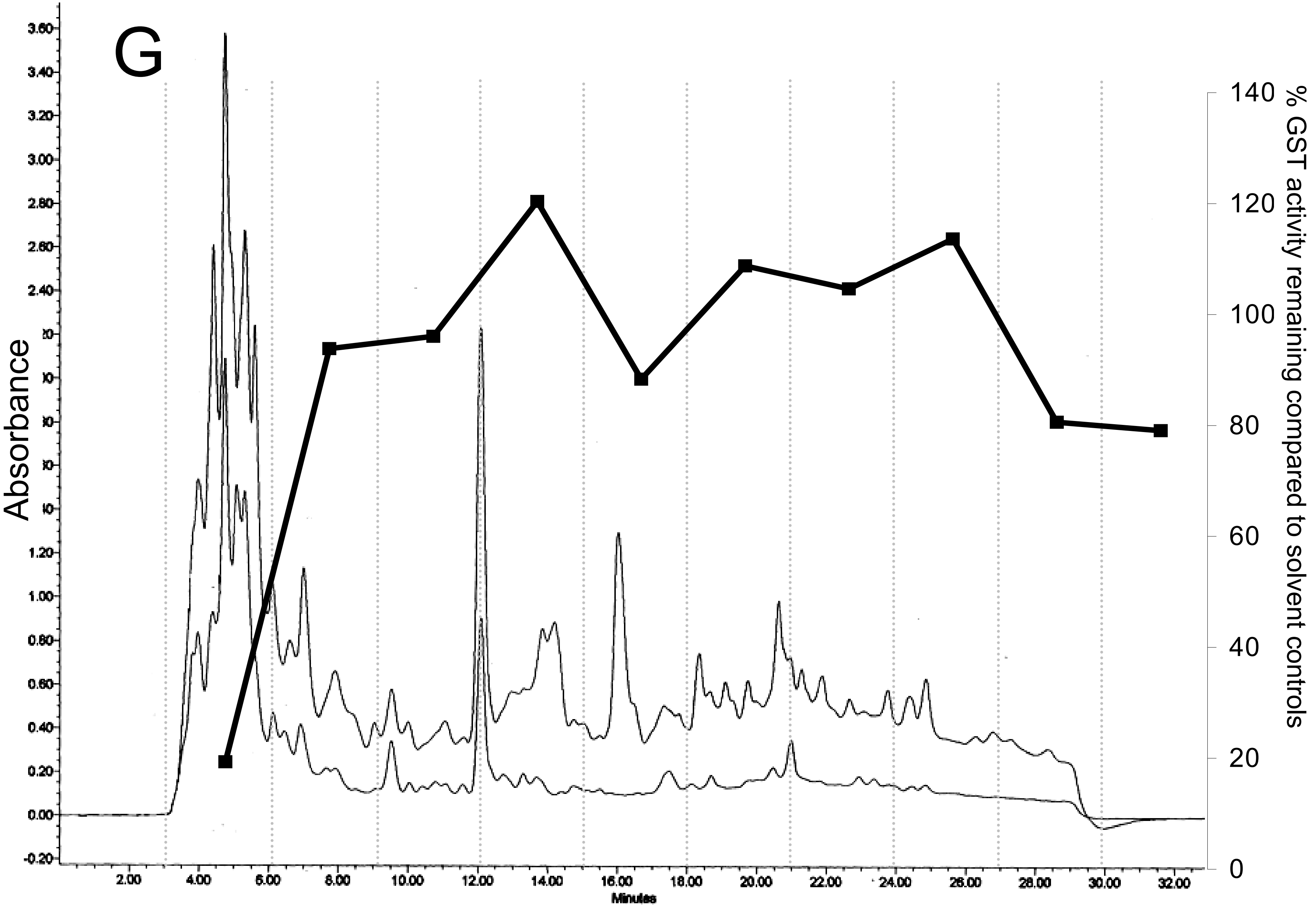

P. elisabethae

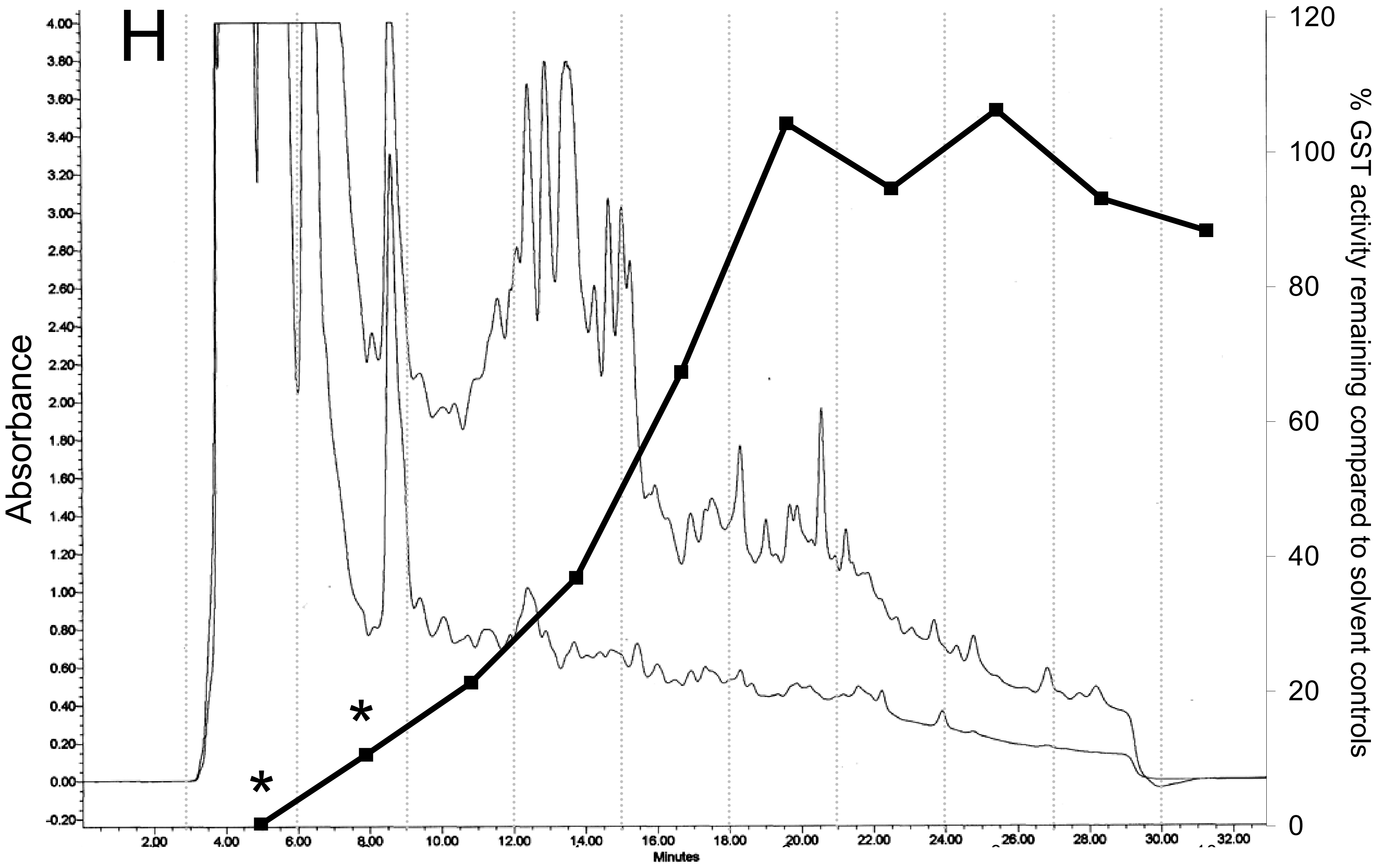
H
Absorbance
% GST activity remaining compared to solvent controls
Minutes
4.00
3.80
3.60
3.40
3.20
3.00
2.80
2.60
2.40
2.20
2.00
1.80
1.60
1.40
1.20
1.00
0.80
0.60
0.40
0.20
0.00
-0.20
2.00
4.00
6.00
8.00
10.00
12.00
14.00
16.00
18.00
20.00
22.00
24.00
26.00
28.00
30.00
32.00
120
100
80
60
40
20
0
*
*

P. homomalla
