# Supplementary material for: Biochemical Warfare on the Reef: The Role of Glutathione Transferases in Consumer Tolerance of Dietary Prostaglandins
Source: PLoS One. 2010 Jan 6;5(1):e8537. doi: 10.1371/journal.pone.0008537 (PMC2796389; doi:10.1371/journal.pone.0008537)

(A) Prostaglandin $A_2$ LC-MS standard (2150 µg injected), $ESI^-$ mode

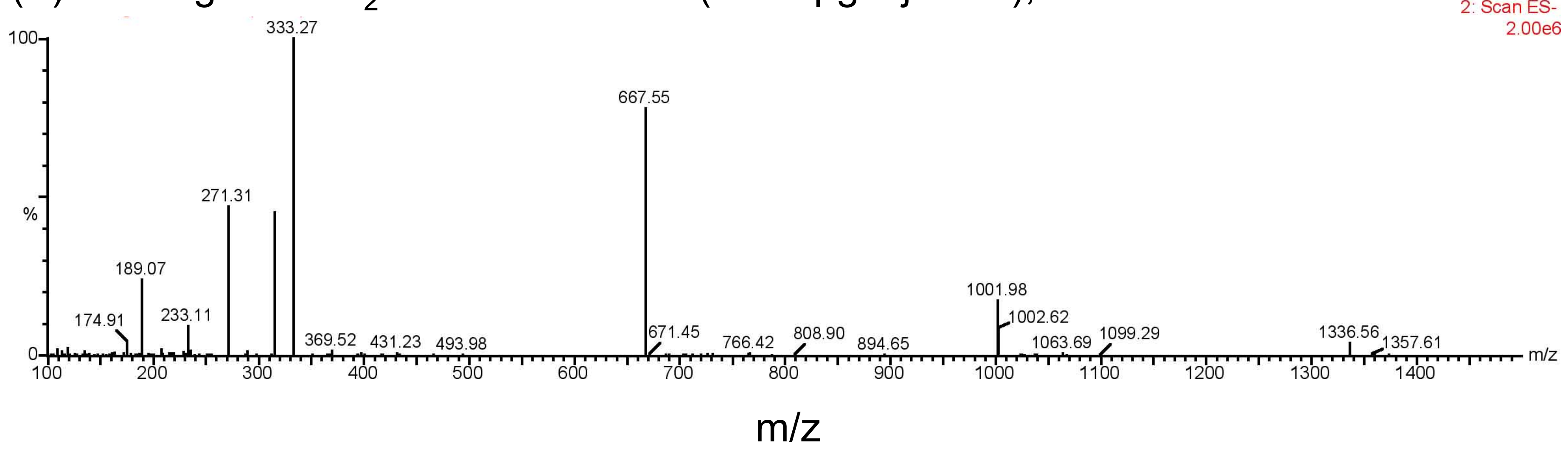


(B) HPLC Fraction 1 (3-6 min, 0.3 ml equv.) from *P. homomalla*, $ESI^-$ mode

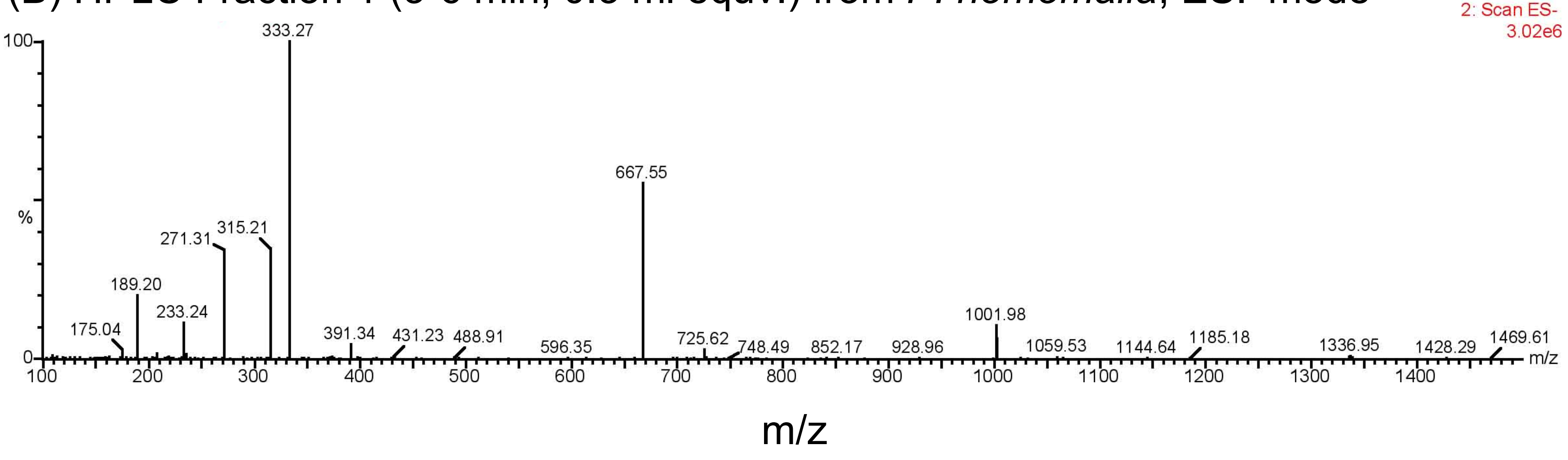

Supplement: Figure S5 — LC-MS spectra of (A) PGA2 standard and (B) HPLC fraction 1 from P. homomalla. (0.34 MB PDF) [file pone.0008537.s005.pdf]
