# Supplementary material for: Biochemical Warfare on the Reef: The Role of Glutathione Transferases in Consumer Tolerance of Dietary Prostaglandins
Source: PLoS One. 2010 Jan 6;5(1):e8537. doi: 10.1371/journal.pone.0008537 (PMC2796389; doi:10.1371/journal.pone.0008537)
Supplement: Figure S2 — Effect of snail diet on GST isoform expression.(A) Bars represent the mean (±SD; n = 39 snails) percent GST subunit composition in affinity-purified digestive gland preparations as a function of snail diet. (B) Bars represent the mean (±SD; n = 39 snails) HPLC peak area normalized for the amount of GST protein applied to the HPLC column as a function of snail diet. (0.35 MB PDF) [file pone.0008537.s002.pdf]

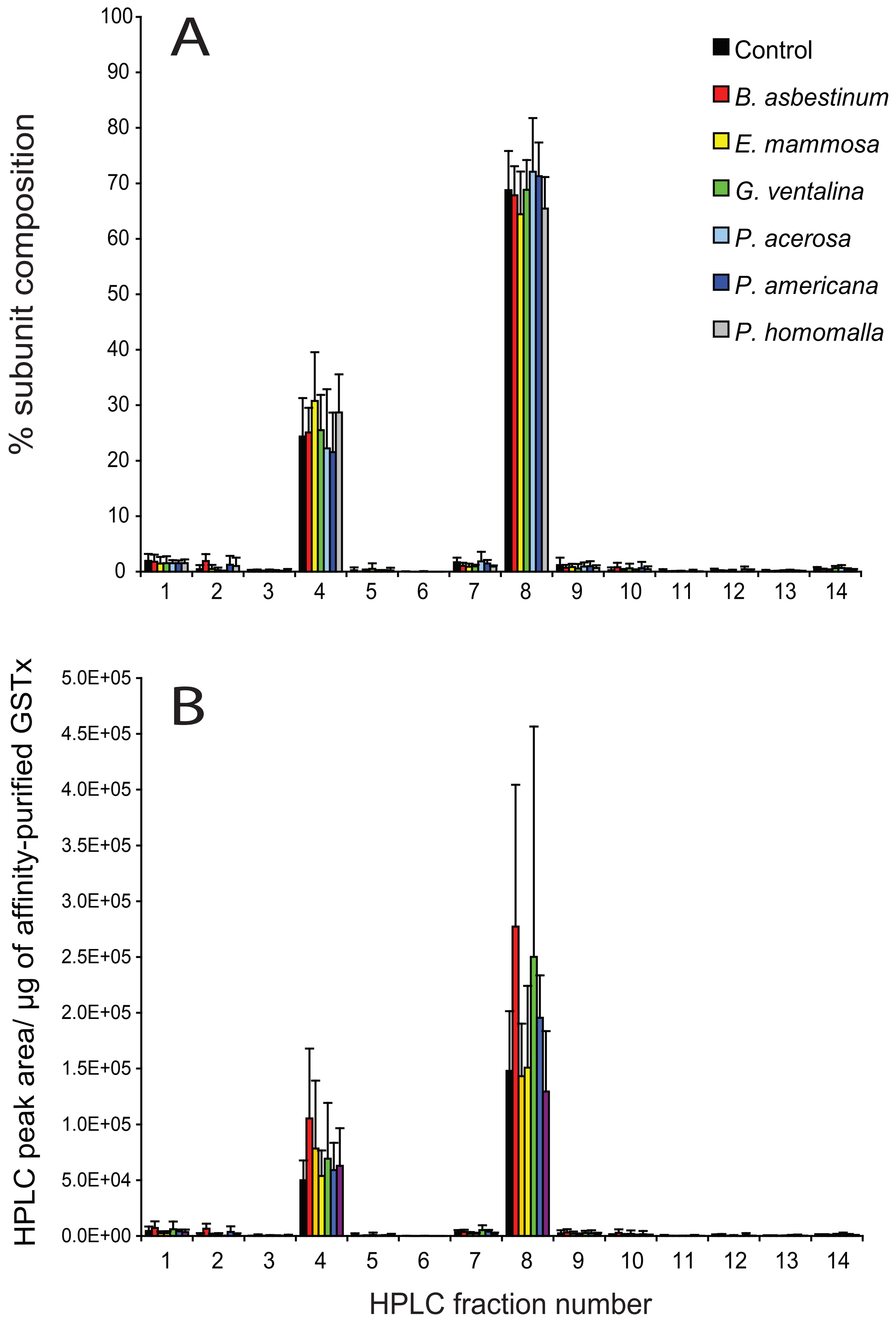

A
B
Control
B. asbestinum
E. mammosa
G. ventalina
P. acerosa
P. americana
P. homomalla
% subunit composition
HPLC peak area/ µg of affinity-purified GSTx
HPLC fraction number
0
10
20
30
40
50
60
70
80
90
100
0.0E+00
5.0E+04
1.0E+05
1.5E+05
2.0E+05
2.5E+05
3.0E+05
3.5E+05
4.0E+05
4.5E+05
5.0E+05
1 2 3 4 5 6 7 8 9 10 11 12 13 14
